# Membrane remodelling triggers maturation of excitation–contraction coupling in 3D-shaped human-induced pluripotent stem cell-derived cardiomyocytes

**DOI:** 10.1007/s00395-023-00984-5

**Published:** 2023-03-29

**Authors:** Fatemeh Kermani, Matias Mosqueira, Kyra Peters, Enrico D. Lemma, Kleopatra Rapti, Dirk Grimm, Martin Bastmeyer, Magdalena Laugsch, Markus Hecker, Nina D. Ullrich

**Affiliations:** 1 Department of Cardiovascular Physiology, Heidelberg University, Heidelberg, Germany; 2 Zoological Institute, Cell and Neurobiology, Karlsruhe Institute of Technology (KIT), Karlsruhe, Germany; 3 Department of Infectious Diseases/Virology, Section Viral Vector Technologies, Heidelberg University, Heidelberg, Germany; 4 German Center for Cardiovascular Research (DZHK), Partner Site Heidelberg/Mannheim, Heidelberg, Germany; 5 German Center for Infection Research (DZIF), Partner Site Heidelberg, Heidelberg, Germany; 6 Institute of Biological and Chemical Systems–Biological information processing (IBCS-BIP), Karlsruhe Institute of Technology (KIT), Eggenstein-Leopoldshafen, Germany; 7 Research Bridge (Synthetic Biology), Heidelberg-Karlsruhe Research Partnership (HEiKA), Heidelberg University and Karlsruhe Institute of Technology, Heidelberg, Germany; 8 Institute of Human Genetics, Heidelberg University, Heidelberg, Germany; 9 Present Address: Department of Engineering, Università Campus Bio-Medico di Roma, Rome, Italy

**Keywords:** hiPSC cardiomyocytes, 3D reshaping, t-tubules, BIN1, Maturation, Excitation–contraction coupling

## Abstract

The prospective use of human-induced pluripotent stem cell-derived cardiomyocytes (hiPSC-CM) for cardiac regenerative medicine strongly depends on the electro-mechanical properties of these cells, especially regarding the Ca^2+^-dependent excitation–contraction (EC) coupling mechanism. Currently, the immature structural and functional features of hiPSC-CM limit the progression towards clinical applications. Here, we show that a specific microarchitecture is essential for functional maturation of hiPSC-CM. Structural remodelling towards a cuboid cell shape and induction of BIN1, a facilitator of membrane invaginations, lead to transverse (t)-tubule-like structures. This transformation brings two Ca^2+^ channels critical for EC coupling in close proximity, the L-type Ca^2+^ channel at the sarcolemma and the ryanodine receptor at the sarcoplasmic reticulum. Consequently, the Ca^2+^-dependent functional interaction of these channels becomes more efficient, leading to improved spatio-temporal synchronisation of Ca^2+^ transients and higher EC coupling gain. Thus, functional maturation of hiPSC-cardiomyocytes by optimised cell microarchitecture needs to be considered for future cardiac regenerative approaches.

**Supplementary Information:**

The online version contains supplementary material available at 10.1007/s00395-023-00984-5.

## Introduction

The technology to generate cardiomyocytes from human-induced pluripotent stem cells (hiPSC-CM) represents a great breakthrough for pharmacological tests and disease modelling in cardiovascular research [12, 22] and fuels new hope for cell-based therapy of patients with heart failure [28, 42]. Human iPSC-CM reveal all essential cardiac features at the electro-mechanical level but retain a premature phenotype compared to ventricular cardiomyocytes of the adult heart. In particular, cell morphology and subcellular microarchitecture remain underdeveloped, lacking the typical anisotropic cuboid shape of adult ventricular cardiomyocytes and the establishment of transverse-axial (t)-tubular network (aka TATS) of the sarcolemma [29, 46]. As a consequence, myofibrils are distributed in a diffuse manner across the rather polygonal cell shape leading to underdeveloped sarcomeres and inefficient contraction [33, 58]. Moreover, the absence of t-tubules, which provide the structural basis for functional interaction between the sarcolemmal L-type Ca^2+^ channel (LTCC) and the intracellular Ca^2+^ release channel, the type 2 ryanodine receptor (RyR2), located at the sarcoplasmic reticulum (SR), is the reason for inefficient Ca^2+^ handling in hiPSC-CM [2, 7]. In adult cardiomyocytes, efficient functional coupling between LTCC and RyR2 is ensured by close proximity of both channels of about 20 nm, a distance that is provided by the dyadic cleft between the t-tubular membrane and the SR [15, 49, 50]. Depolarization-induced activation of LTCCs leads to Ca^2+^ influx at the t-tubular membrane, which triggers further Ca^2+^ release by RyR2s needed for contraction, a process called Ca^2+^-induced Ca^2+^ release (CICR) [5]. The efficiency of this excitation–contraction (EC) coupling mechanism is fundamentally dependent on the amplification of the initial Ca^2+^ influx by RyR2-mediated Ca^2+^ release and on the spatio-temporal synchronisation of this Ca^2+^ release throughout the cell, which is normally ensured by the well-organised t-tubular network deep into the cell [4, 9]. Absence or disruption of the t-tubular network may alter CICR leading to a reduction of the EC coupling gain, as known from cardiomyocytes derived from hypertrophied or failing heart [47, 57]. In hiPSC-CM, such a well-structured tubular network and consequently the formation of dyads are not yet developed causing abnormal and inefficient Ca^2+^ handling and EC coupling [61]. So far, the triggers for the development of t-tubules in premature cardiomyocytes have not yet been identified, and many approaches have been tested to drive these novel cardiomyocytes to further structural and functional maturation in vitro. They comprise biophysical and biochemical stimulations [36, 45, 59], co-cultures with cardiac non-cardiomyocytes [8, 19], cell or substrate patterning and tissue engineering [27, 40, 43, 46, 51].

Recent research has focussed intensively on the investigation of the effect of topographical and physical cues on the maturation of hiPSC-CM during culture. Cardiomyocytes grown on micropatterned substrates enhanced cell alignment and improved Ca^2+^ handling and contraction [23, 35, 40, 54]. In a recent study, we reported that single mouse iPSC-CM seeded into cuboid 3D micro-scaffolds produced strong tubular invaginations and revealed more robust Ca^2+^ signalling [46].

Apart from environmental parameters, the question arises which cellular mechanisms may be involved in tubulogenesis. Few proteins have come into the focus of attention as potential key players in the process of t-tubule and dyad formation, specifically caveolin-3, nexilin and junctophilin. While overexpression of caveolin-3 prevented the loss of tubular network in cardiomyocytes from pressure overload-induced failing mouse hearts [24], loss of nexilin, a component of cardiac dyads, resulted in a loss of the tubular network and disruption of dyads in ventricular cardiomyocytes [30]. Junctophilin-2 expression may be involved in t-tubule development and dyad stabilisation in hiPSC-CM [10, 37].

Here, we focus on the potential role of the bridging integrator-1 protein (BIN1) in t-tubule biogenesis [3, 14, 17]. As a member of the BAR (BIN1-amphiphysin/Rvs) domain superfamily, BIN1 is involved in multiple cellular processes including membrane trafficking, recycling and remodelling, cytoskeleton regulation, muscle development, DNA repair and apoptosis [39]. BIN1 has been identified in the brain, heart and skeletal muscle, and is composed of: (i) an amino-terminal BAR domain, which oligomerizes and forms a “banana-shaped” molecule to interact with plasma membrane lipids; (ii) a tissue-specific proline-rich middle linkage domain; and (iii) a carboxy-terminal SH3 domain interacting with intracellular proteins [15]. BIN1 has not only been shown to be indispensable for muscle development, t-tubule formation, Ca^2+^ homeostasis and fibre organisation of skeletal muscle cells [38, 52], but a reduction of BIN1 has also been described in diseased cardiomyocytes, thereby correlating with a disruption of the t-tubular network [18, 55].

To induce further maturation of hiPSC-CM, we merged two approaches and combined 3D reshaping together with BIN1 expression to trigger structural remodelling and maturation. We tested the hypothesis that reshaping hiPSC-CM in pre-designed cuboid 3D micro-scaffolds and overexpression of BIN1 lead to tubulogenesis and improve Ca^2+^ handling at the level of EC coupling. Our data demonstrate that a cardiomyocyte-specific microarchitecture comprising a t-tubular-like membrane network is required for efficient EC coupling in hiPSC-CM and, thus, for the development of robust and mature cardiomyocytes. Our findings open new avenues for future hiPSC-cardiomyocytes development and applications as cardiac cell grafts.

## Methods

### Human iPS cells and cardiogenic differentiation

Human iPS cell lines were kindly provided by Dr. Lukas Cyganek, Stem Cell Unit Göttingen, University Medical Center Göttingen (UMG). Wild-type iPSC line UMGi014-C clone 14 (isWT1.14) was generated from dermal fibroblasts using the integration-free Sendai virus and described previously [44]. Two other cell lines were used to replicate the representative experiments (ethical approval number for all hiPSC lines used in this study: S-455/2018). hiPSC were differentiated into cardiomyocytes by modulation of the Wnt/β-catenin signalling pathway at 95–100% confluency as previously described [62]. After differentiation, cells were purified by metabolic selection using glucose-free and sodium–lactate-supplemented medium to prevent growth of non-cardiomyocytes. Finally, cells were maintained in RPMI plus B27 with insulin until further experiments. A detailed description of the differentiation and purification methods is provided in the Supplemental files.

### General methods

Detailed methods for cardiomyocyte preparation from hiPSC and culture, 3D micro-scaffold production, SEM imaging, AAV6-mediated transduction, immunocytochemistry, PLA, live Ca^2+^ imaging, cellular electrophysiology, quantitative PCR, Western blotting and statistical analysis are provided in the Supplemental file section.

## Results

### BIN1 is successfully expressed in transduced hiPSC-CM

**Fig. 1:**
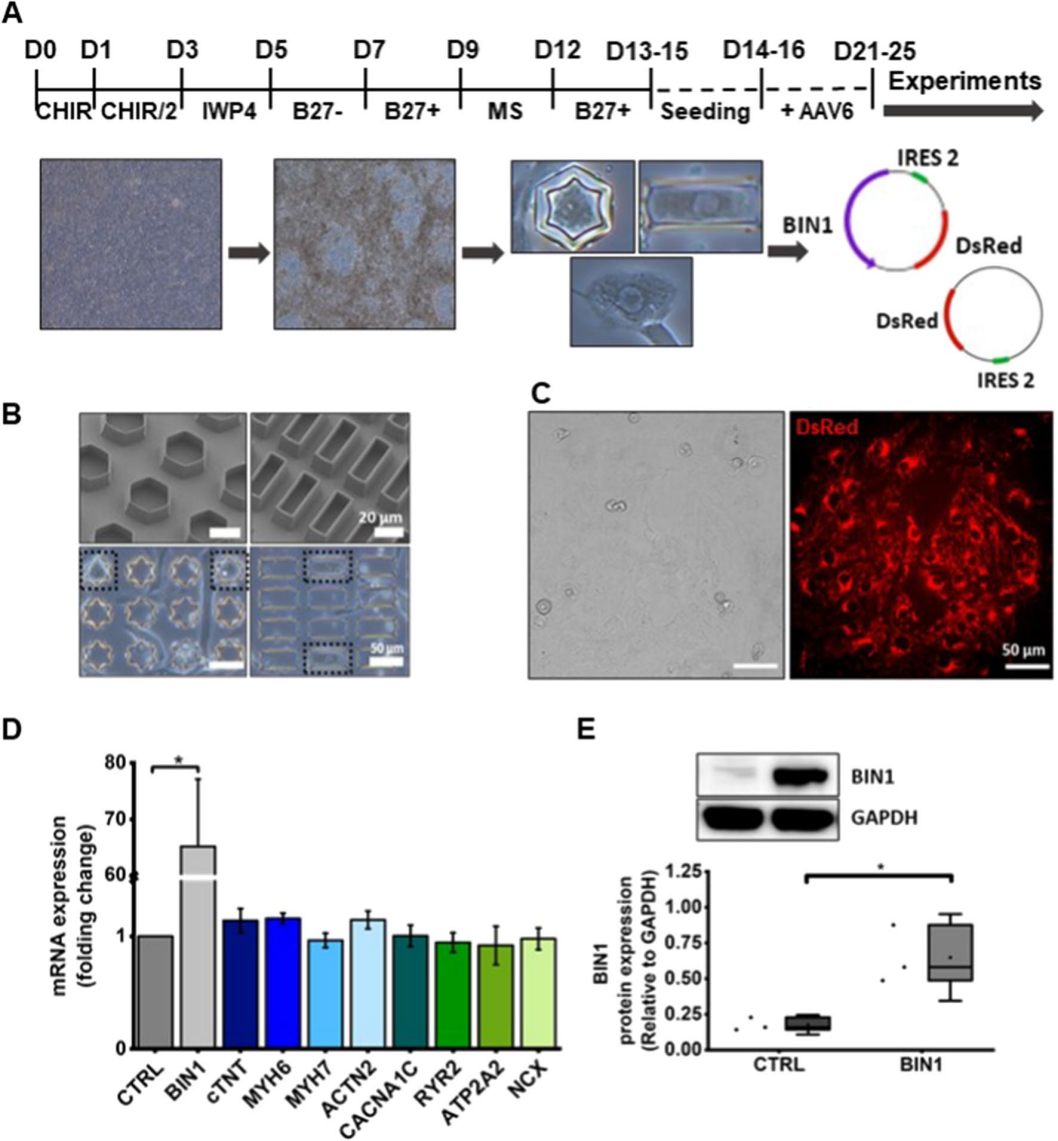
BIN1 is successfully expressed in transduced hiPSC-CM. **A** Schematic timeline of cardiogenic differentiation and subsequent experimental design. **B** 3D scaffolds in hexagonal (left images) and cuboid (right images) shapes. **C** Representative images of bright field (left image) and dsRed expression (right image) of hiPSC-CM. **D** Quantification of mRNA expression levels relative to control, measured by real-time qRT-PCR (*N* = 4–5, *p < 0.05 for BIN1 vs. CTRL). The list of primers can be found in Table S1. **E** Immunoblot of BIN1 protein expression in transduced hiPSC-CM. Quantification of the change in protein abundance of BIN1 (*N* = 3, *p < 0.05). Data are presented as mean ± SE (bar graph) or mean ± SD (box blot), significance tested by Student’s *t* test

To test our hypothesis whether 3D reshaping induced structural remodelling, single hiPSC-CM were plated on a planar surface and in 3D micro-scaffolds with hexagonal and cuboid shapes. To recapitulate the rectangular structure of adult cardiomyocytes, 3D-cuboid scaffolds were designed accordingly to provide a long axis and spatial restrictions to further grow in height as described before [46]. Hexagonal 3D scaffolds with similar surface area were employed for direct comparison of cells growing in 3D-confined structures without cell elongation [46] (Fig. 1A, B). Adherent cells were transduced with an Adeno-associated virus 6 (AAV6) vector carrying BIN1 and a fluorescent marker (dsRed), or a control vector containing only dsRed. After 5–6 days of transduction, cells were utilised for further experiments. As shown in Fig. 1C, hiPSC-CM revealed strong levels of red fluorescence confirming successful transduction. BIN1 expression was significantly boosted at both mRNA (*p* = 0.005) and protein (*p* = 0.017) levels in hiPSC-CM transduced with BIN1 compared to control, which only showed low levels of BIN1 protein (Fig. 1D, E). We also assessed the effect of BIN1 overexpression on the mRNA expression levels of proteins of the contractile apparatus, including myofilaments like cardiac troponin T (cTNT), myosin heavy chain 6 (MYH6), MYH7 and α-actinin (ACTN2), and Ca^2+^-handling proteins such as the L-type Ca^2+^ channel (CACNA1C), RYR2, SERCA2 (ATP2A2) and Na^+^/Ca^2+^ exchanger (NCX). The mRNA levels of these targets remained unaffected by BIN1 overexpression.

### 3D reshaping and BIN1 overexpression lead to adaptations in cell morphology and microarchitecture

**Fig. 2:**
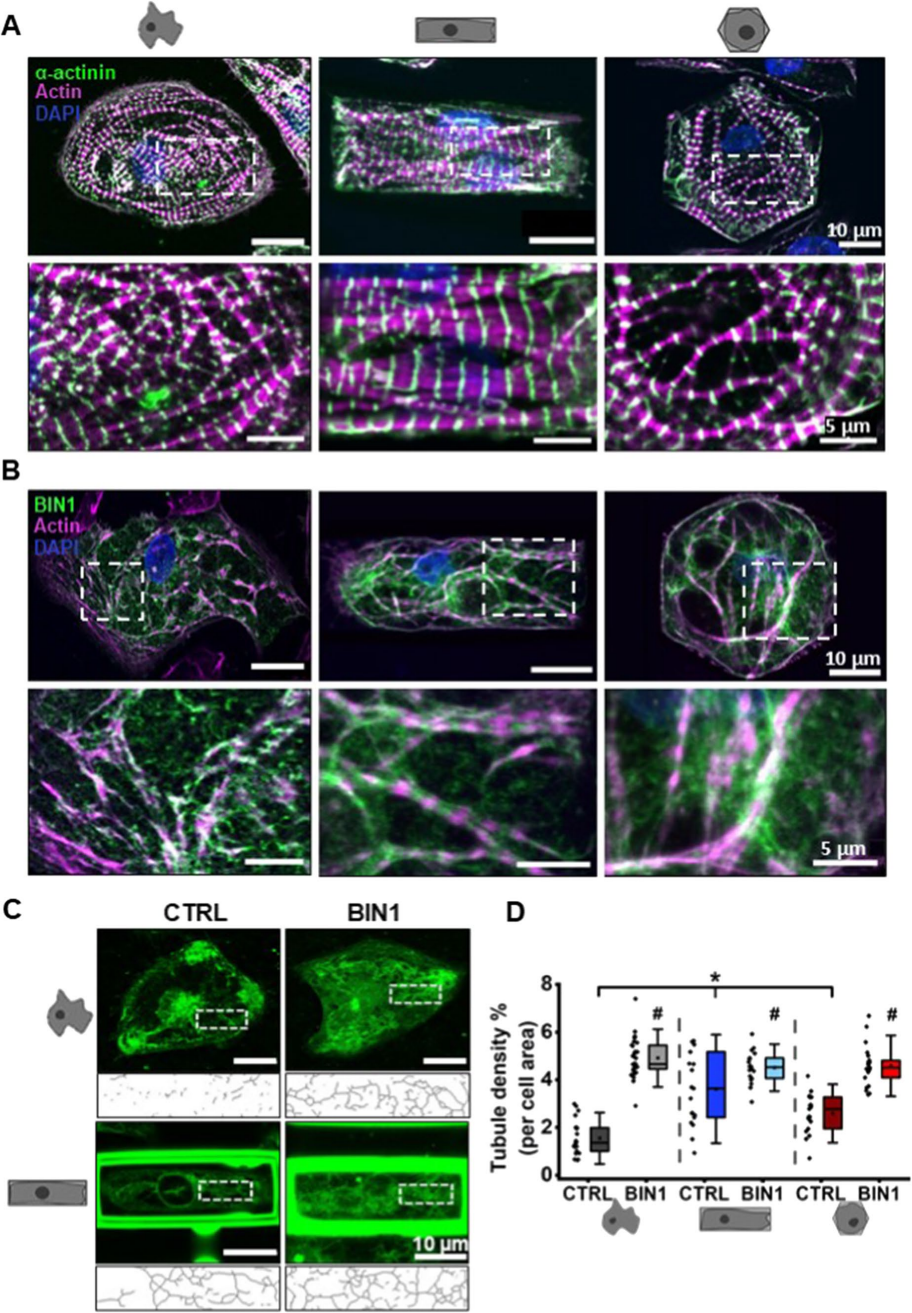
3D reshaping and BIN1 overexpression lead to adaptations in cell morphology and microarchitecture. Immunolabeling of α-actinin (green, **A**), BIN1 (green, **B**), actin (magenta) and DNA (DAPI, blue) in BIN1-expressing, non-patterned, cuboid and hexagonally shaped hiPSC-CM. White boxes indicate the area of magnification. **C** Representative images of confocal live cell imaging of the sarcolemma using di-8-ANEPPS to visualise the tubular membrane network. White boxes illustrate the skeletonized images of the analysed regions of interest (ROI). **D** Statistical analysis of the tubule density within the ROIs in different experimental groups of hiPSC-CM. Two-way ANOVA was conducted to examine the synergistic effect of BIN1 expression and 3D reshaping on tubule density. *N* = 4, *n* = 19–31 cells; *indicates comparison between different shapes, and # between BIN1-overexpressing and shape control cells; *p* < 0.05. Data are presented as a box plot and whiskers show SD

In comparison to rod-shaped adult ventricular cardiomyocytes, which present parallel-aligned and organised myofibrils, 2D-cultured hiPSC-CM are rather flat with no defined long axis formation. To assess the shape of hiPSC-CM and myofibril structures in 3D micro-scaffolds, the organisation of the sarcomeric α-actinin and actin was investigated. As shown in Fig. 2A, non-patterned hiPSC-CM revealed a round shape with irregular and random orientation of actin filaments throughout the cell. The distribution pattern of myofilaments in hexagonally shaped hiPSC-CM was also similar to non-patterned cells. Conversely, hiPSC-CM grown in cuboid micro-scaffolds displayed an elongated anisotropic shape with well-organised myofibrils and parallel-aligned α-actinin, indicating sarcomeric units. This remarkable alignment of myofibrils along the long axis of the cell in a cuboid scaffold demonstrates the great power of reshaping to induce subcellular remodelling processes in hiPSC-CM.

In the next step, we investigated the synergistic potency of 3D reshaping and BIN1 overexpression on triggering the generation of membrane invaginations forming t-tubules, which are critical for proper EC coupling. Immunolabeling of BIN1 confirmed the expression of BIN1 in transduced hiPSC-CM presenting strong sarcolemmal invaginations indicative of the development of an early but still unstructured tubular network (Fig. 2B, Figures SIA, B). In comparison, hiPSC-CM transduced with the control vector did not reveal any substantial levels of BIN1 expression nor plasma membrane invaginations (Figure SIC). For further detailed investigations of the origin of the tubular network, cells were recorded using the fluorescent membrane dye di-8-ANEPPS in confocal live imaging experiments. Since this dye only incorporates within the plasma membrane, but not in subcellular membrane compartments of intact cells, fluorescence signals of tubular structures derived exclusively from plasma membrane invaginations. The confocal images showed extensive tubular network generation in 3D-reshaped BIN1-overexpressing hiPSC-CM. Moreover, statistical analysis confirmed that there was a significant dual effect of 3D reshaping and BIN1 overexpression on increasing tubule density as assessed by two-way ANOVA (F(2, 125) = 20.034, *p* < 0.05; Fig. 2C, D). Interestingly, 3D reshaping without BIN1 overexpression also elicited remarkable tubule generation compared to control (non-patterned cells), but with high variability (Fig. 2D). Adding BIN1 to it significantly enhanced t-tubule formation and thereby reduced this large variability in all groups. So, the results demonstrate a combined effect of reshaping and BIN1 expression with regard to t-tubule density. We further confirmed that both 3D reshaping and BIN1 overexpression significantly induced tubular membrane network in hiPSC-CM derived from another stem cell line with a different genetic background (Figure SII).

### Membrane remodelling reorganises the expression pattern of Ca^2+^ handling proteins and induces dyad formation

**Fig. 3:**
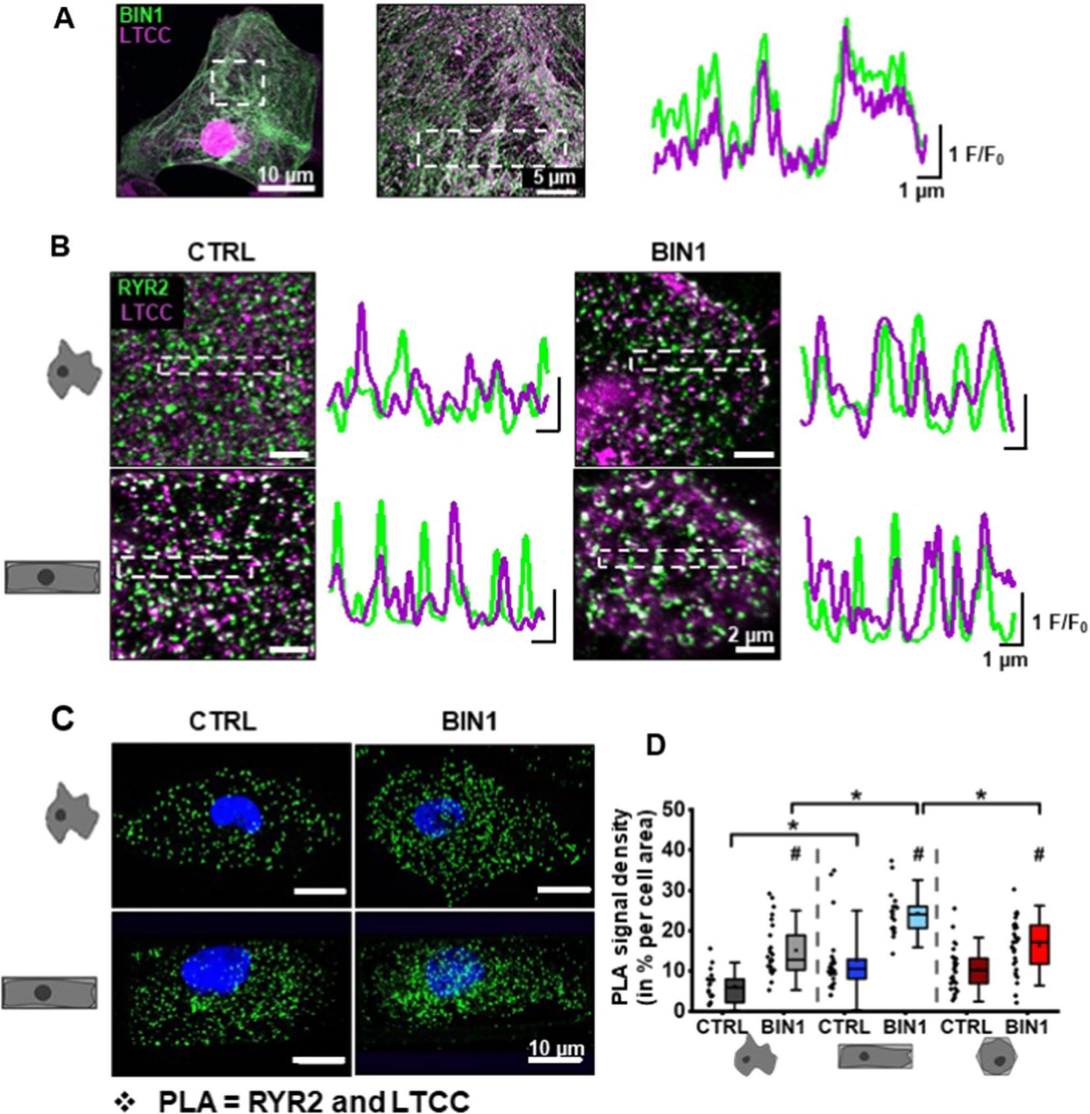
Membrane remodelling reorganises the expression pattern of Ca^2+^ handling proteins and induces dyad formation. **A** Representative confocal images of the expression pattern of BIN1 (green) and LTCC (magenta) in a BIN1-expressing hiPSC-CM (left image; centre image: magnification of the ROI) and intensity profiles of the depicted ROI in the centre image. **B** Immunolabelling of RYR2 (green) and LTCC (magenta) and intensity profiles from the ROIs (white boxes) demonstrating spatial alignment of both ion channels relative to each other. **C** Representative confocal images from PLA: green signals indicate the sites of interaction between RYR2 and LTCC in dyad microdomains (< 40 nm). Nuclei are stained in blue. **D** PLA signal density was measured as a fraction of cell area occupied by fluorescence signals. Two-way ANOVA was conducted to examine the effect of BIN1 expression and 3D reshaping on LTCC-RyR2 cluster density. *N* = 4, *n* = 18–30 cells; * indicates comparison between different shapes; # indicates comparison between BIN1-overexpressing and shape control cells; *p* < 0.05. Data are presented as a box plot and whiskers show SD

Since the close proximity of LTCCs and RYR2s is vital for efficient CICR and EC coupling, in the next step, we investigated the influence of 3D reshaping and BIN1-induced membrane invaginations on the expression pattern of these Ca^2+^ channels. Immunolabeling of BIN1 and LTCC illustrated that BIN1 enhanced LTCC clustering along membrane tubules (Fig. 3A). This notion is further supported by the overlapping fluorescence signal peaks in the merged line profiles (right panel). Moreover, double staining of RYR2s and LTCCs manifested spatial clustering of these proteins relative to each other. The line profiles of both Ca^2+^ channels showed a higher degree of fluorescent signal overlap in cuboid BIN1-overexpressing hiPSC-CM as compared to control non-patterned and hexagonally shaped cells (Fig. 3B, Figure SIIIA). In line with this experiment, we performed highly specific and sensitive proximity ligation assays (PLA) to further verify the close localization of LTCCs and RYR2s within the BIN1-induced vicinity of t-tubules and SR. Both 3D reshaping and BIN1 overexpression had a significant effect on the density of closely located LTCCs and RyR2s in hiPSC-CM; however, statistically seen, there was no interaction between all shapes as assessed by two-way ANOVA (F(2, 139) = 2.598, *p* = 0.078; Fig. 3C, D, Figure SIIIB). Detailed analysis showed that PLA signal density was significantly higher in cuboid, BIN1-expressing hiPSC-CM compared to non-patterned BIN1-expressing and cuboid control hiPSC-CM. This suggests that BIN1 expression together with rectangular reshaping of hiPSC-CM may have an additive effect on augmenting the probability of forming dyadic structures. Overall, these results support the concept that BIN1 may serve as a local anchor for stabilisation of LTCCs and RYR2s in close proximity in t-tubular and SR membranes to trigger the formation of functional dyads in hiPSC-CM, promoting co-localization and further functional interaction.

### Characterisation of I_CaL_ properties in reshaped BIN1-overexpressing hiPSC-CM

**Fig. 4:**
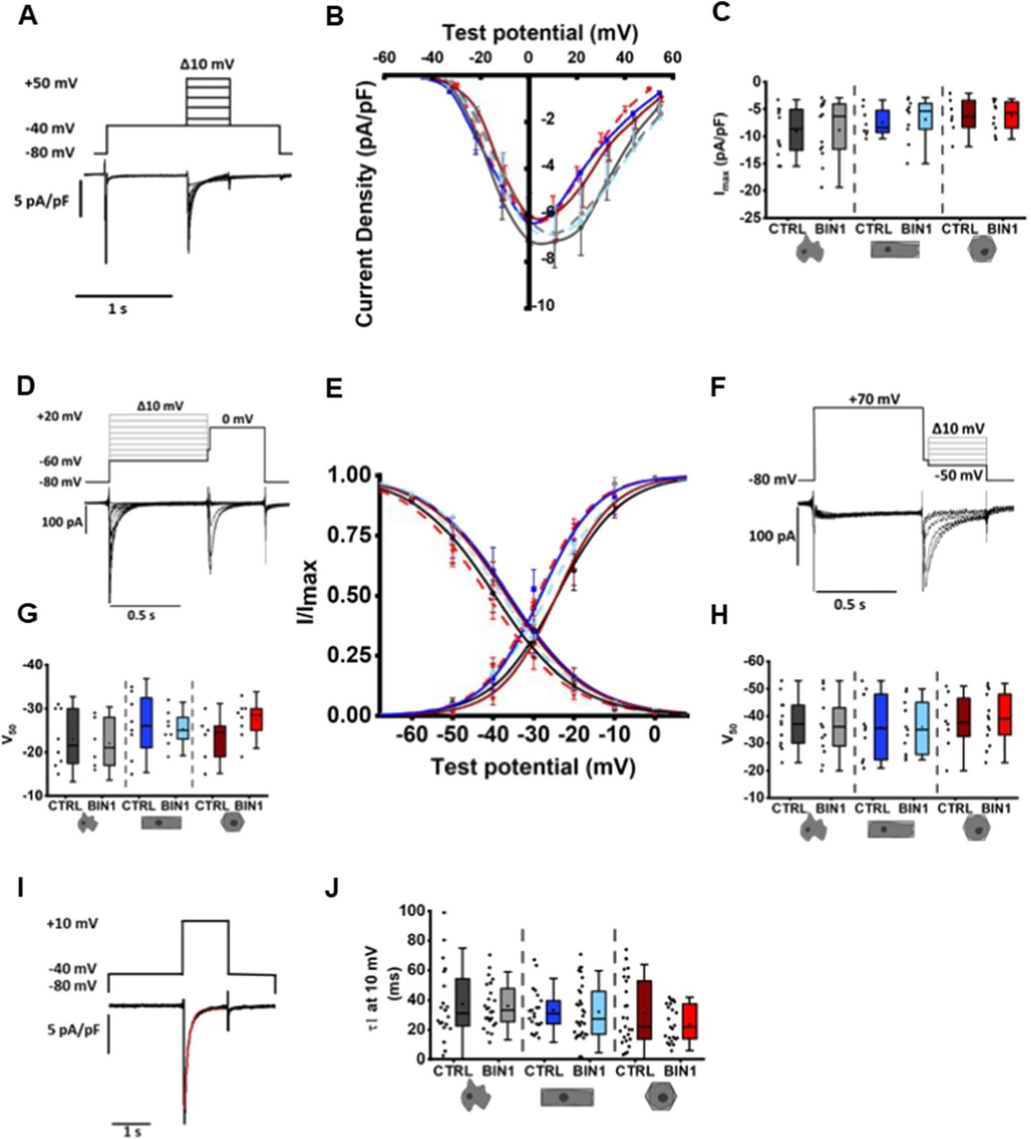
Characterisation of I_CaL_ properties in reshaped BIN1-overexpressing hiPSC-CM. **A**, **D**, **F**, **I** Representative I_CaL_ traces triggered by the indicated voltage protocols. **B** Current–voltage relationship, **E** voltage-dependent steady-state activation and inactivation curves. **C** Analysis of peak current (I_max_, *N* = 4–5, *n* = 7–13 cells). **G, H** Analysis of half-maximal voltage-dependent (V_1/2_) activation and inactivation (*N* = 4–5, *n* = 6–13 cells). **I, J** Analysis of the time constant of the Ca^2+^-dependent inactivation of I_CaL_ (τ1) at + 10 mV fitted with a bi-exponential function (*N* = 7–8, *n* = 20–32 cells). Two-way ANOVA revealed no significant differences between the experimental groups. Data are presented as a box plot and whiskers show SD

The spatial rearrangement of LTCCs and RyR2s fuels the assumption that enhanced dyad formation may have functional consequences for the EC coupling mechanism. To investigate Ca^2+^ handling in reshaped and BIN1-overexpressing cells, we first characterised Ca^2+^ influx via LTCCs in more detail. Single hiPSC-CM were patch clamped in the whole-cell configuration and membrane Ca^2+^ currents (I_CaL_) were measured using the indicated voltage clamp protocols. In each cell, the current–voltage (IV) relationship, the voltage dependence of activation and the voltage dependence of inactivation were examined with independent voltage protocols. From the IV curve, the current amplitudes were measured to assess the peak Ca^2+^ current amplitudes in dependence of the clamped membrane potential. Total membrane current was then normalised to cell capacitance to obtain the LTCC current density per cell, a measure to compare the different values of different cells and cell sizes. Figure 4A illustrates the IV curves of I_CaL_ recorded from hiPSC-CM of the different experimental groups. On average, the peak I_CaL_ amplitudes at different potentials were not significantly different in the experimental groups. This finding was further confirmed by measuring and comparing the voltage-dependent activation and inactivation states of I_CaL_. From the steady-state activation and inactivation curves (Fig. 4D–F), the half-maximal voltages for activation and inactivation were derived (Fig. 4G and H). Both values are required from each cell to characterise the LTCC gating kinetics. As expected, our data demonstrate that V_1/2_ of I_CaL_ activation and inactivation of the different experimental groups were not statistically different from each other. To investigate the fast Ca^2+^-dependent inactivation of I_CaL_, the first time constant (τ1) of current decay measured at + 10 mV was compared among the different experimental groups (Figs. 4F, G). Again, no significant differences were detected.

### Characterisation of Ca^2+^ spark events in reshaped BIN1-overexpressing hiPSC-CM

**Fig. 5:**
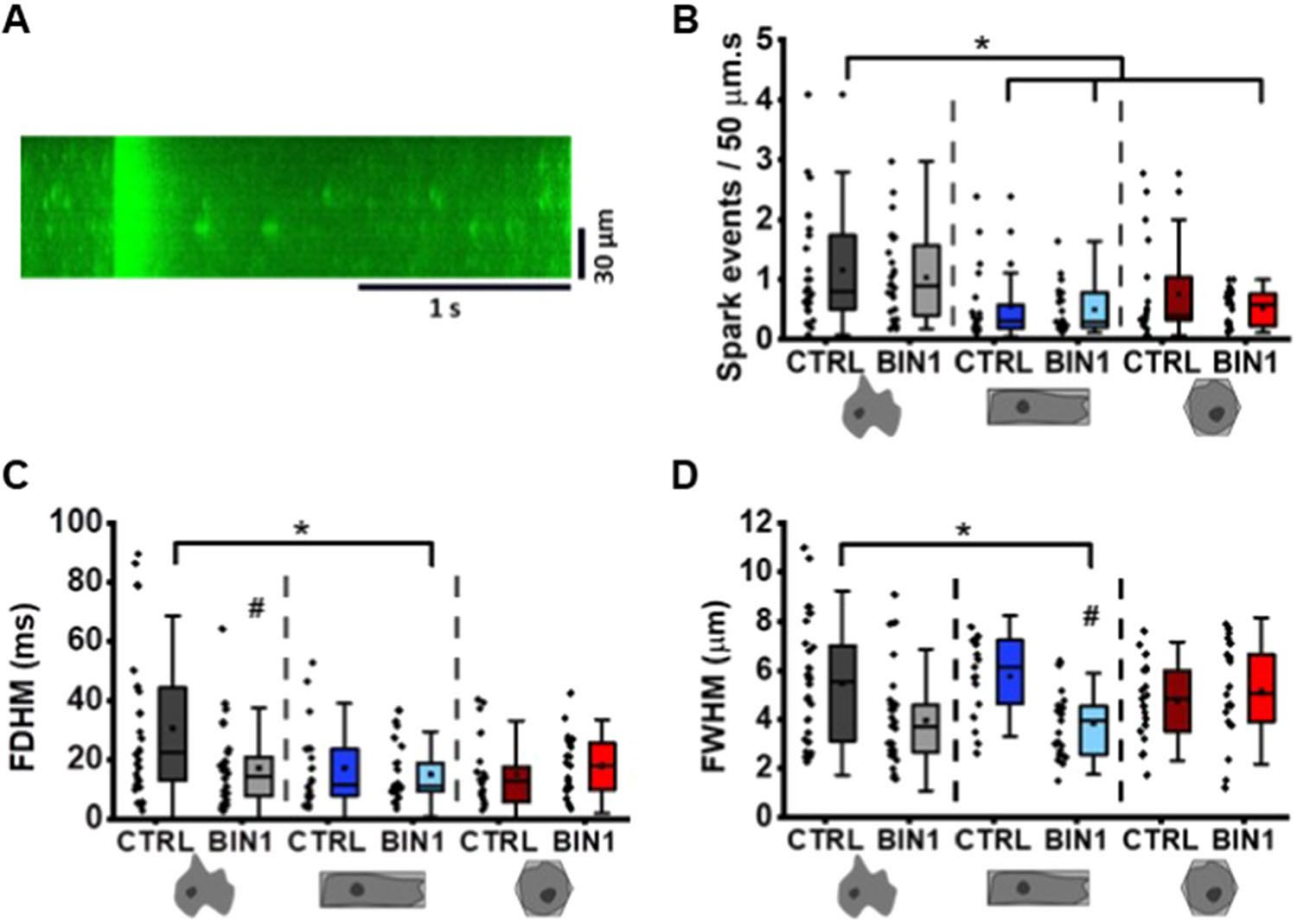
Characterisation of Ca^2+^ spark events in reshaped BIN1-overexpressing hiPSC-CM. **A** Representative line-scan image of a spontaneous Ca^2+^ transient and Ca^2+^ sparks. Analysis of the number of spark events per 50 µm and second (**B**), full duration at half maximum (FDHM, **C**) and full width at half maximum (FWHM, **D**) in the different groups of hiPSC-CM. Statistical differences were tested by two-way ANOVA (*N* = 3, *n* = 17–30 cells); *indicates comparison between different shapes; # indicates comparison between BIN1-overexpressing and shape control cells; *p* < 0.05. Data are presented as a box plot and whiskers show SD

Since I_CaL_ properties were unchanged in the different experimental groups, we took a closer look at RyR2 function. To this end, we measured spontaneous Ca^2+^ release events and characterised the spatial and temporal properties of Ca^2+^ sparks. Spontaneous Ca^2+^ sparks were recorded in the confocal line-scan mode using the Ca^2+^-sensitive dye fluo-4 (Fig. 5A). Interestingly, 3D reshaping of hiPSC-CM resulted in a significant reduction of spark frequency (Fig. 5B) and spark duration (Fig. 5C). In addition, BIN1 overexpression significantly reduced spark width (Fig. 5D). Two-way ANOVA revealed a synergistic effect of 3D reshaping and BIN1 overexpression on the temporal (FDHM: F(2, 132) = 3.534, *p* = 0.032) and spatial dynamics (FWHM: F(2, 131) = 3.401, *p* = 0.036) of the Ca^2+^ sparks.

### Maturation of Ca^2+^ transient dynamics in 3D-reshaped BIN1-overexpressing hiPSC-CM

**Fig. 6:**
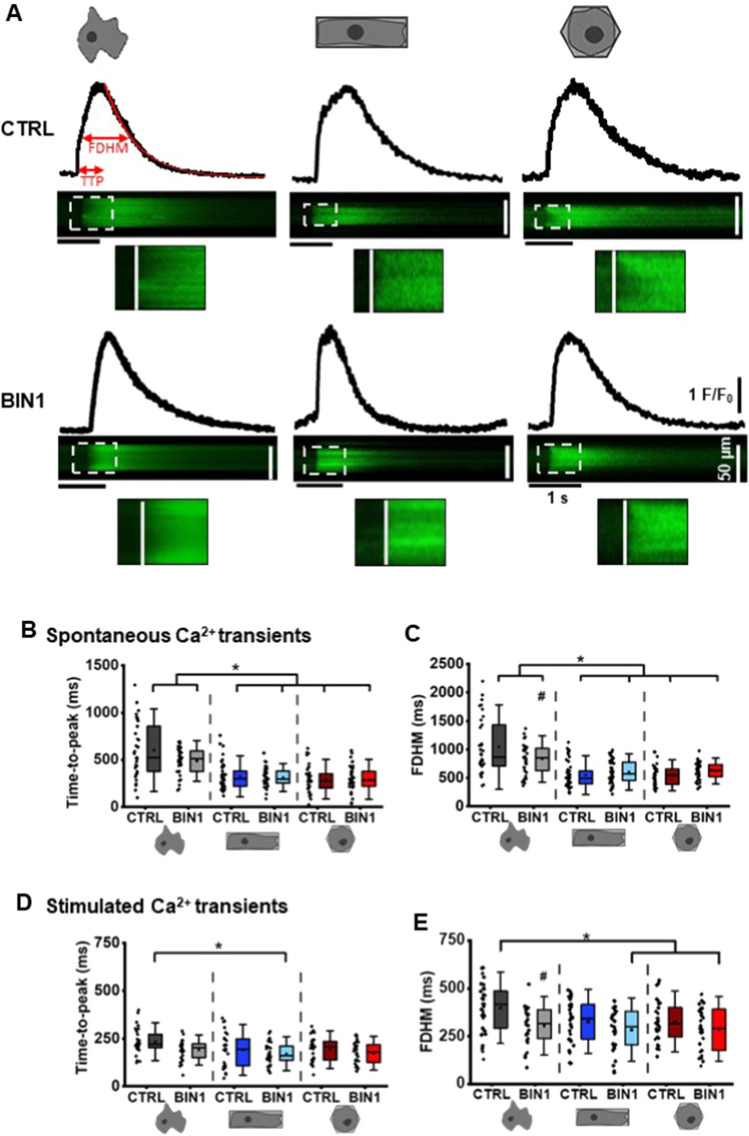
Maturation of Ca^2+^ transient dynamics in 3D-reshaped BIN1-overexpressing hiPSC-CM. **A** Representative line-scan images and plot profiles of spontaneous Ca^2+^ transients in the different experimental groups. Analysis of time-to-peak (TTP; **B, D**), and full duration half maximum (FDHM; **C, E**) of spontaneous and stimulated Ca^2+^ transients, respectively. Statistical differences were tested by two-way ANOVA (*N* = 3–4, *n* = 33–49 cells); *indicates comparison between different shapes; #indicates comparison between BIN1-overexpressing and shape control cells; *p* < 0.05. Data are presented as a box plot and whiskers show SD

Having shown that 3D reshaping and BIN1 overexpression elicit structural remodelling, we, in turn, tested our hypothesis that structural remodelling also favours functional adaptations. To examine this concept, we recorded spontaneous Ca^2+^ transients in confocal line-scan imaging using the Ca^2+^-sensitive fluorescent indicator fluo-4. Representative line-scan images and line profiles of each experimental group are summarised in Fig. 6A. Detailed analysis of Ca^2+^transient kinetics revealed that 3D reshaping significantly reduces time-to-peak (TTP, *p* < 0.001, Fig. 6B) and decay times (*p* < 0.001, Fig. 6C), while BIN1 overexpression and 3D reshaping significantly interact to decrease the time of full duration at half-maximal Ca^2+^ transient amplitude (FDHM, F(2, 222) = 8.77, *p* < 0.001, Fig. 6D) in the hiPSC-CM. We further confirmed that both 3D reshaping and BIN1 overexpression also significantly accelerated spontaneous Ca^2+^ transients at the level of Ca^2+^ release and reuptake in the second hiPSC-CM line (Figure SIV). Interestingly, cuboid BIN1-overexpressing hiPSC-CM revealed the best synchronised Ca^2+^ transients compared to any other conditions whereas remarkable delays of Ca^2+^ release were observed in the corresponding control groups (Fig. 6A, please refer to the zoomed images). In line with these observations, time-to-peak (TTP, Fig. 6E), decay (Fig. 6F) and FDHM (Fig. 6G) of stimulated Ca^2+^ transients were also significantly shortened in 3D-reshaped BIN1-overexpressing hiPSC-CM, particularly in the cuboid cell group.

### Excitation–contraction coupling gain is improved in cuboid BIN1-overexpressing hiPSC-CM

**Fig. 7:**
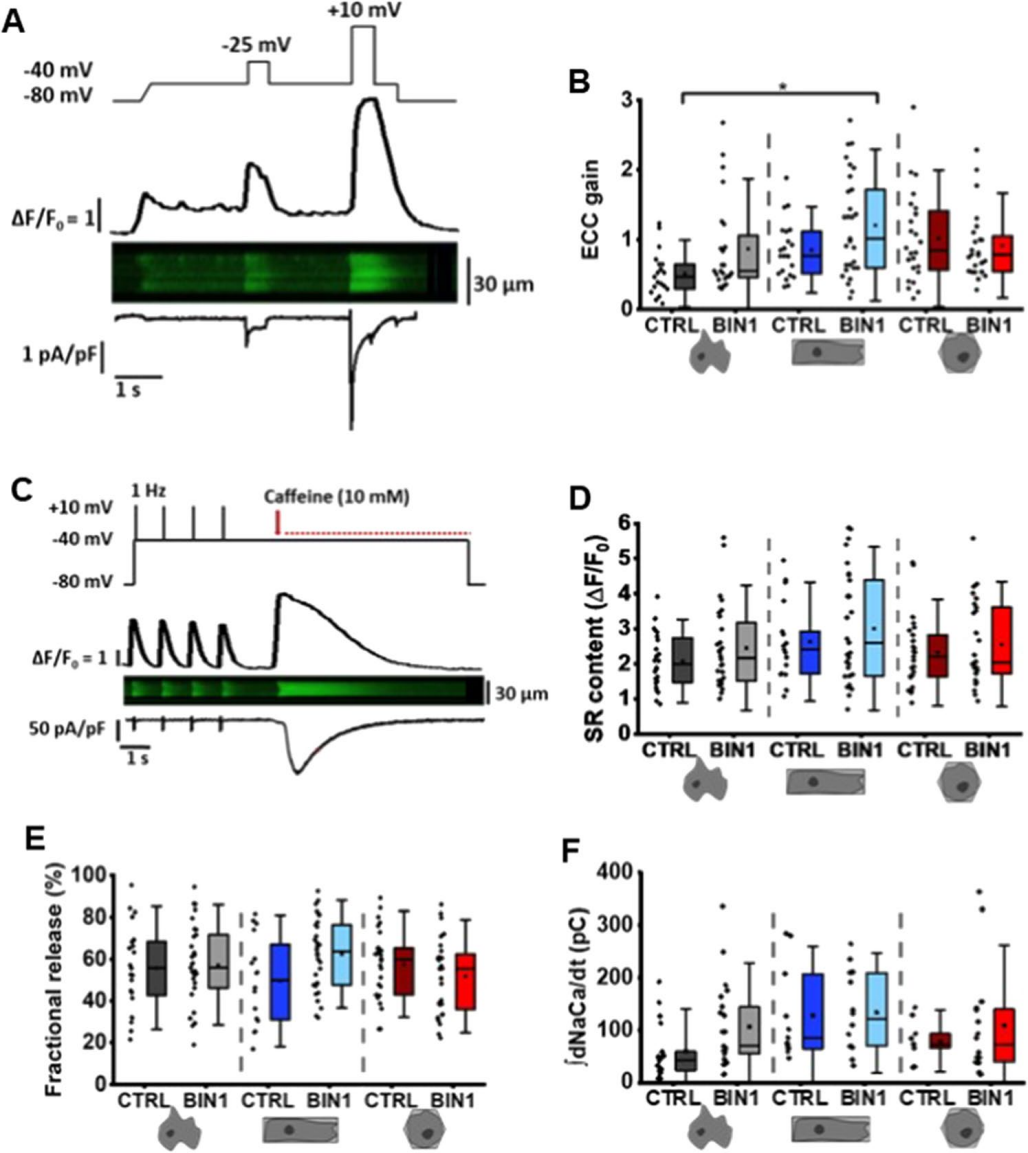
EC coupling gain is improved in cuboid BIN1-expressing hiPSC-CM. **A** Voltage protocol for I_CaL_ stimulation (upper panel), Ca^2+^ transients with corresponding line-scan image and line profile (middle panel) and recordings of I_CaL_ (lower traces). **B** EC coupling gain measured at − 25 mV (*N* = 7–8, *n* = 19–29 cells) and distribution curves. **C** Stimulation protocol (upper panel), Ca^2+^ transient (line-scan and line profile, middle panel), and current traces (lower traces) to measure fractional release. Evaluation of **(D)** SR content, **E** fractional release and **F** NCX activity in hiPSC-CM (*N* = 5–6, *n* = 9–24 cells). Statistical comparison by two-way ANOVA. Data are presented as a box plot and whiskers show SD. *indicates comparison between different shapes; # indicates comparison between BIN1-overexpressing and control cells; *p* < 0.05

Since we observed that Ca^2+^ transients were faster in 3D-reshaped BIN1-overexpressing hiPSC-CM, we also investigated EC coupling in more detail by analysing the EC coupling gain. This gain provides an assessment of the coupling fidelity between LTCCs and RyR2s. L-type Ca^2+^ currents (I_CaL_) and current-stimulated Ca^2+^ transients were measured simultaneously with the following stimulation protocol: I_Na_ was inactivated by a voltage ramp from the holding potential (V_H_) of − 80 mV to − 40 mV, then Ca^2+^ release was triggered by two separate voltage steps to − 25 mV and + 10 mV (please see voltage protocol in Fig. 7A). The EC coupling gain was calculated from the ratio of the peak Ca^2+^ transient amplitude and the corresponding peak I_CaL_ at − 25 mV. The maximal I_CaL_ and Ca^2+^ release amplitude at + 10 mV were measured for internal control. Interestingly, in 3D-reshaped and BIN1-overexpressing hiPSC-CM, distribution of the EC coupling gain moved towards higher values as compared to control non-patterned hiPSC-CM indicating improvement of coupling and, therefore, more efficient CICR (Fig. 7B). Moreover, two-way ANOVA revealed that both 3D reshaping and BIN1 overexpression have statistically significant effects on the EC coupling gain; however, no significant synergistic effects were observed (F(2, 135) = 2.435, *p* = 0.091, Fig. 7B). In conclusion, these experiments demonstrate that BIN1-overexpressing hiPSC-CM grown in cuboid micro-scaffolds develop better EC coupling leading to a significantly larger amplification of the I_CaL_-triggered Ca^2+^ release compared to non-patterned control cells (Fig. 7B).

Finally, dependence of Ca^2+^ release from the filling state of the SR was determined in 1 Hz-stimulated patch-clamped cells. Fractional release of Ca^2+^ from the SR was determined by normalising the peak amplitude of paced Ca^2+^ transients to the peak amplitude of caffeine-induced Ca^2+^ transients (Fig. 7C, D). At constant triggering, on average 50% of the SR Ca^2+^ content was released per twitch, indicating similar Ca^2+^ release properties and SR Ca^2+^ loads in the different groups of hiPSC-CM (Fig. 7D, E). This was further reflected by similar activities of the NCX in these cells assessed as integrated inward membrane currents (∫I_NCX_) during prolonged caffeine application (Fig. 7F). The expression pattern of the two major proteins for Ca^2+^ removal, i.e. the reuptake of Ca^2+^ into the SR via SERCA2 and the extrusion of Ca^2+^ through the NCX, in non-patterned and 3D-reshaped BIN1-overexpressing hiPSC-CM is shown in Figure SV. SERCA2 expression revealed a dense network lining sarcolemmal BIN1 expression (Figure SVA) indicative of a tight association of the SR and the sarcolemma in BIN1-overexpressing hiPSC-CM. In contrast, NCX staining revealed a dotted distribution pattern over the entire sarcolemma (Figure SVB).

A comparison of the presented data is summarised in Table 1 to illustrate the specific effects of shape (cuboid/hexagon), BIN1 expression and combined effects on hiPSC-CM structure and function compared to non-patterned control cells.

**Table 1:**
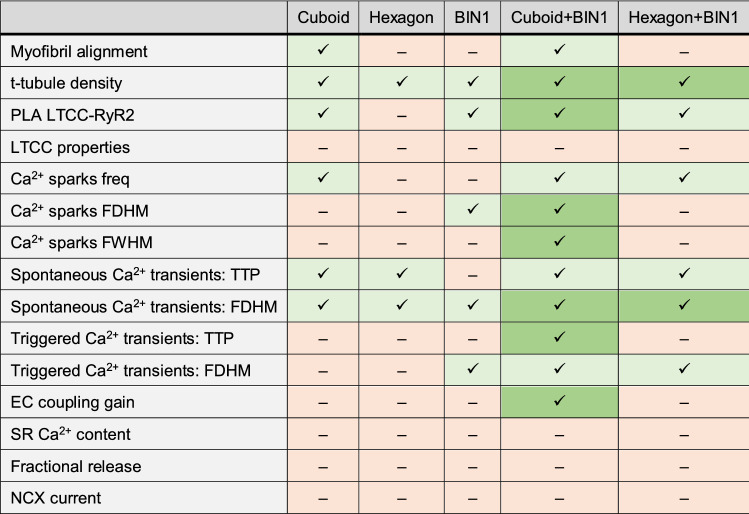
Summary of data comparison and illustration of additive effects: red boxes represent no significant differences compared to non-patterned control, light green boxes indicate statistically significant effects, dark green boxes indicate additive effects of particular shape and BIN1 expression

## Discussion

Despite their cardiogenic properties, hiPSC-CM differ from adult cardiomyocytes in many structural and functional details, which are generally summarised and connoted as immature features. The term immaturity in this context is inspired and derived from the immature characteristics of prenatal or neonatal cardiomyocytes. Despite distinct differences in the development, differentiation and properties of native or stem cell-derived cardiomyocytes, the concept of immaturity conveys the idea that, in compliance with native cells, further maturation of hiPSC-CM towards an adult phenotype is possible. Since the immaturity of hiPSC-CM limits their application for clinical purposes, current research focuses intensively on the development of efficient maturation strategies to achieve an adult-like phenotype that may be best suited for a cell-based therapy of the failing heart.

In this study, we focussed on the combined effects of changes in cell shape towards more adult morphology and molecular enhancement of BIN1 expression on Ca^2+^ handling and, thus, the basis of EC coupling in hiPSC-CM. In line with previous work [46], our data demonstrate that 3D reshaping induces structural reorganisation at the level of the subcellular microarchitecture in hiPSC-CM. Cuboid cells display parallel-aligned myofibrils, well-organised sarcomeres and a tubular membrane network reminiscent of early t-tubules. Since hiPSC-CM not only attach to the bottom surface but also to the walls of the scaffolds, resulting in an increase in cell height [46], the additional mechanical strain on the cell membrane may play a role in t-tubule biogenesis in 3D-reshaped hiPSC-CM, for which the triggering mechanism remains to be elucidated. In this context, passive resistance of the walls of the scaffold in conjunction with membrane stretch during contraction may activate mechanosensitive pathways such as, e.g. ion channels of the TRP family [56], piezo channels [21, 60] and/or integrin-dependent outside-in signalling pathways [20] to elicit specific gene programmes for membrane remodelling.

In addition to morphological remodelling by changing cell shape, we also overexpressed BIN1 in the hiPSC-CM. Previous reports suggested a possible role of BIN1 in the generation of membrane tubular networks [14, 41, 52]. Our findings demonstrate a strong induction of membrane invaginations by BIN1 overexpression already in non-patterned control hiPSC-CM, and even more so in cuboid cells, revealing a new level of membrane remodelling. Since BIN1 serves as a local anchor for LTCCs to stabilise their expression and localization at t-tubules [17], t-tubule formation is strictly followed by a subcellular rearrangement of LTCCs and RyR2s. The localization of LTCCs and RyR2s switched from a diffuse distribution to a more organised and striated expression pattern in cuboid BIN1-overexpressing hiPSC-CM leading to increased formation of dyads, i.e. subcellular microdomains where LTCCs and RyR2s are enriched for better functional interaction [11, 3]. In contrast to non-patterned control cells, the distance between both types of Ca^2+^ channel was reduced to less than 40 nm in 3D-reshaped BIN1-overexpressing hiPSC-CM, reminiscent of the narrow dyadic cleft of adult, i.e. mature cardiomyocytes. This close vicinity is indispensable for the functional interaction of LTCCs and RyR2s via rapid diffusion of Ca^2+^ ions, thus ensuring efficient EC coupling.

One hallmark of immaturity in hiPSC-CM is their spontaneous contractile activity. Although varied mechanisms underlying this autorhythmicity have been hotly debated, the interplay between so-called membrane and Ca^2+^ clock mechanisms may provide a plausible explanation. At the molecular level, hiPSC-CM expressed all essential Ca^2+^ handling proteins contributing to Ca^2+^ influx, release and removal [61] to a similar degree in non-patterned and 3D-reshaped cells. While the membrane clock depends on the alternating activity of sarcolemmal ion channels, spontaneous Ca^2+^ release from the SR is key to the Ca^2+^ clock mechanism [26, 34, 53]. Therefore, we measured Ca^2+^ currents and both spontaneous and Ca^2+^-triggered Ca^2+^ release events, respectively.

Electrophysiological evaluation of the LTCC-dependent Ca^2+^ current revealed no difference in its properties and kinetics among the different groups of hiPSC-CM, but Ca^2+^ signalling differed at the functional level pointing to a more mature Ca^2+^ handling in the cytoplasm. One indicator for this in structurally remodelled hiPSC-CM was the reduction in spontaneous Ca^2+^ release events from the SR. While non-patterned control cells revealed a high frequency of Ca^2+^ sparks and related spontaneous Ca^2+^ release events at resting conditions, their occurrence was significantly decreased in cuboid BIN1-overexpressing hiPSC-CM. Detailed analysis of spontaneous and Ca^2+^-triggered Ca^2+^ release events revealed faster Ca^2+^ transients in 3D-remodelled cells irrespective of the shape both at the level of Ca^2+^ release from the SR (TTP) and Ca^2+^ reuptake via SERCA2 and extrusion via the NCX (FDHM), which is in accordance with previous studies of either BIN1-overexpressing or 3D-reshaped iPSC-CM [16, 46].

In dysfunctional cardiomyocytes originating from different models of cardiac disease [1, 13], loss of the t-tubular network and significantly decreased dyadic units are highly prevalent. Moreover, cardiomyocytes in which t-tubule abundance is reduced reveal a decline in both BIN1 and LTCC abundance and significantly delayed Ca^2+^ transients [16]. Furthermore, the maladaptive remodelling processes leading to heart failure generally result in abnormal Ca^2+^ handling and specifically in desynchronised Ca^2+^ transients in the affected cardiomyocytes with the consequence of severely reduced EC coupling and decreased contractile force [32, 57]. Hence, a well-structured tubular membrane network and the formation of functional dyads are essential for robust Ca^2+^ handling in cardiomyocytes, and therefore represent a major goal to achieve in hiPSC-CM [29]. In non-patterned control hiPSC-CM, the early phase of the Ca^2+^ transient, which is initiated by diffusion-dependent activation of the RyR2s via Ca^2+^ influx through the LTCCs, revealed strong desynchronization at the onset of the Ca^2+^ transients. This pattern can be best explained by inward propagation of the released Ca^2+^ from the peripheral cell membrane towards the cell centre rather than true CICR. In contrast, cuboid BIN1-expressing hiPSC-CM demonstrated a spatio-temporally synchronised onset of Ca^2+^ release from the SR. Due to the many BIN1-induced membrane invaginations, LTCCs are also located deep in the centre of the cell. As a consequence, sarcolemmal depolarization and LTCC-mediated Ca^2+^ influx lead to local CICR via junctional RyR2s simultaneously throughout the entire cell and not only at the cell periphery.

This improvement of junctional CICR was further corroborated by the significantly enhanced EC coupling gain in cuboid 3D-reshaped, BIN1-overexpressing hiPSC-CM. Our data demonstrate that despite similar levels of expression of the Ca^2+^ channels and Ca^2+^ influx currents, Ca^2+^ release from the SR was significantly greater in these cardiomyocytes due to shorter Ca^2+^ diffusion distances and, thus, a more effective EC coupling gain. This increased gain is an important measure for amplification of the cytosolic Ca^2+^ signal through RyR2-mediated Ca^2+^ release from the SR and, therefore, confirms that structural remodelling leads to robust EC coupling, indicative of important functional maturation processes at the level of single hiPSC-CM.

In summary, our data provide strong evidence that structural remodelling of hiPSC-CM at the level of cell morphology and membrane organisation leads to optimization of the functional interaction between LTCCs and RyR2s, the key players of EC coupling in cardiomyocytes. In this context, the role of BIN1 as important steering wheel for membrane invaginations and consequently for the development of a more mature Ca^2+^ handling machinery suggests that BIN1 may also represent a promising target for the treatment of heart failure. Potential therapeutic effects of BIN1 gene therapy have already been tested at the preclinical level with preliminary success [31, 63]. With regard to hiPSC-CM-based cell replacement therapy, the combination of cardiomyocyte-specific BIN1 induction and tissue engineering approaches employing micropatterning of cells [6] may advance the development of cardiac patches and enhance the therapeutic potential of these cells for the treatment of patients with heart failure.

## Limitations of the study

Here, we have addressed different levels of structural remodelling in the context of the rather complex development of fully mature cardiomyocytes from pluripotent stem cells that is in addition accompanied by some technical limitations. Our experimental model served primarily the identification of specific cues and features that are needed to drive hiPSC-CM to further maturation. In this context, one significant success in the maturation process is already a reduction in variability, as seen from our data. In the presented form, the tool of reshaping is not suited for in vivo experimentation, but importantly, the gain of knowledge that both longitudinal shape and t-tubule formation are required for functional maturation, will have to be considered for the preparation of cell grafts. In future studies, the impact of the composition of the extracellular matrix (ECM) and its stiffness [25] as well as the effect of outside-in signalling [23] on the functional maturation of these cells must be taken into account. More physiological growth surfaces may also permit prolonged culture times and, thus, a more effective functional maturation [48] of these cells. Therefore, it will be interesting to expand our newly developed experimental model for reshaping single cells and analysing EC coupling properties to multicellular hiPSC-CM preparations with similar cellular characteristics. Instead of BIN1 overexpression by molecular engineering, it will be interesting to find more natural mechanisms to enhance endogenous BIN1 levels not only for the functional maturation of hiPSC-CM but also for the treatment of diseased adult cardiomyocytes with reduced t-tubules and EC coupling.

### Supplementary Information

Below is the link to the electronic supplementary material.

Supplementary file1 (DOCX 765 KB)

## Data Availability

All data generated or analyzed during this study are included in this published article and its supplementary information files.

## References

[1] Balijepalli RC, Lokuta AJ, Maertz NA, Buck JM, Haworth RA, Valdivia HH, Kamp TJ (2003). Depletion of T-tubules and specific subcellular changes in sarcolemmal proteins in tachycardia-induced heart failure. Cardiovasc Res.

[2] Brette F, Orchard C (2003). T-tubule function in mammalian cardiac myocytes. Circ Res.

[3] De La Mata A, Tajada S, O’Dwyer S, Matsumoto C, Dixon RE, Hariharan N, Moreno CM, Santana LF (2019). BIN1 induces the formation of T-tubules and adult-like Ca 2+ release units in developing cardiomyocytes. Stem Cells.

[4] Eisner DA, Caldwell JL, Kistamás K, Trafford AW (2017). Calcium and excitation-contraction coupling in the heart. Circ Res.

[5] Fabiato A (1983). Calcium-induced release of calcium from the cardiac sarcoplasmic reticulum. Am J Physiol - Cell Physiol.

[6] Falconnet D, Csucs G, Michelle Grandin H, Textor M (2006). Surface engineering approaches to micropattern surfaces for cell-based assays. Biomaterials.

[7] Franzini-Armstrong C, Protasi F, Ramesh V (1999). Shape, size, and distribution of Ca2+ release units and couplons in skeletal and cardiac muscles. Biophys J.

[8] Giacomelli E, Meraviglia V, Campostrini G, Cochrane A, Cao X, van Helden RWJ, Krotenberg Garcia A, Mircea M, Kostidis S, Davis RP, van Meer BJ, Jost CR, Koster AJ, Mei H, Míguez DG, Mulder AA, Ledesma-Terrón M, Pompilio G, Sala L, Salvatori DCF, Slieker RC, Sommariva E, de Vries AAF, Giera M, Semrau S, Tertoolen LGJ, Orlova VV, Bellin M, Mummery CL (2020). Human-iPSC-derived cardiac stromal cells enhance maturation in 3D cardiac microtissues and reveal non-cardiomyocyte contributions to heart disease. Cell Stem Cell.

[9] Greenstein JL, Hinch R, Winslow RL (2006). Mechanisms of excitation-contraction coupling in an integrative model of the cardiac ventricular myocyte. Biophys J.

[10] Gross P, Johnson J, Romero CM, Eaton DM, Poulet C, Sanchez-Alonso J, Lucarelli C, Ross J, Gibb AA, Garbincius JF, Lambert J, Varol E, Yang Y, Wallner M, Feldsott EA, Kubo H, Berretta RM, Yu D, Rizzo V, Elrod J, Sabri A, Gorelik J, Chen X, Houser SR (2021). Interaction of the joining region in junctophilin-2 with the L-Type Ca2+channel is pivotal for cardiac dyad assembly and intracellular Ca2+dynamics. Circ Res.

[11] Guo J, Tian Q, Barth M, Xian W, Ruppenthal S, Schaefers HJ, Chen Z, Moretti A, Laugwitz KL, Lipp P (2022). Human BIN1 isoforms grow, maintain, and regenerate excitation-contraction couplons in adult rat and human stem cell-derived cardiomyocytes. Cardiovasc Res.

[12] Haupt LP, Rebs S, Maurer W, Hübscher D, Tiburcy M, Pabel S, Maus A, Köhne S, Tappu R, Haas J, Li Y, Sasse A, Santos CCX, Dressel R, Wojnowski L, Bunt G, Möbius W, Shah AM, Meder B, Wollnik B, Sossalla S, Hasenfuss G, Streckfuss-Bömeke K (2022). Doxorubicin induces cardiotoxicity in a pluripotent stem cell model of aggressive B cell lymphoma cancer patients. Basic Res Cardiol.

[13] He JQ, Conklin MW, Foell JD, Wolff MR, Haworth RA, Coronado R, Kamp TJ (2001). Reduction in density of transverse tubules and L-type Ca2+ channels in canine tachycardia-induced heart failure. Cardiovasc Res.

[14] Hong T, Yang H, Zhang SS, Cho HC, Kalashnikova M, Sun B, Zhang H, Bhargava A, Grabe M, Olgin J, Gorelik J, Marbán E, Jan LY, Shaw RM (2014). Cardiac BIN1 folds T-tubule membrane, controlling ion flux and limiting arrhythmia. Nat Med.

[15] Hong TT, Shaw RM (2017). Cardiac t-tubule microanatomy and function. Physiol Rev.

[16] Hong TT, Smyth JW, Chu KY, Vogan JM, Fong TS, Jensen BC, Fang K, Halushka MK, Russell SD, Colecraft H, Hoopes CW, Ocorr K, Chi NC, Shaw RM (2012). BIN1 is reduced and Cav1.2 trafficking is impaired in human failing cardiomyocytes. Heart Rhythm.

[17] Hong TT, Smyth JW, Gao D, Chu KY, Vogan JM, Fong TS, Jensen BC, Colecraft HM, Shaw RM (2010). BIN1 localizes the L-type calcium channel to cardiac T-tubules. PLoS Biol.

[18] Howe K, Ross JM, Loiselle DS, Han JC, Crossman DJ (2021). Right-sided heart failure is also associated with transverse tubule remodeling in the left ventricle. Am J Physiol Heart Circ Physiol.

[19] Huang CY, Peres Moreno Maia-Joca R, Ong CS, Wilson I, DiSilvestre D, Tomaselli GF, Reich DH (2020). Enhancement of human iPSC-derived cardiomyocyte maturation by chemical conditioning in a 3D environment. J Mol Cell Cardiol.

[20] Israeli-Rosenberg S, Manso AM, Okada H, Ross RS (2014). Integrins and integrin-associated proteins in the cardiac myocyte. Circ Res.

[21] Jiang F, Yin K, Wu K, Zhang M, Wang S, Cheng H, Zhou Z, Xiao B (2021). The mechanosensitive Piezo1 channel mediates heart mechano-chemo transduction. Nat Commun.

[22] Jung P, Seibertz F, Fakuade FE, Ignatyeva N, Sampathkumar S, Ritter M, Li H, Mason FE, Ebert A, Voigt N (2022). Increased cytosolic calcium buffering contributes to a cellular arrhythmogenic substrate in iPSC-cardiomyocytes from patients with dilated cardiomyopathy. Basic Res Cardiol.

[23] Kit-Anan W, Mazo MM, Wang BX, Leonardo V, Pence IJ, Gopal S, Gelmi A, Nagelkerke A, Becce M, Chiappini C, Harding SE, Terracciano CM, Stevens MM (2021). Multiplexing physical stimulation on single human induced pluripotent stem cell-derived cardiomyocytes for phenotype modulation. Biofabrication.

[24] Kong CHT, Bryant SM, Watson JJ, Roth DM, Patel HH, Cannell MB, James AF, Orchard CH (2019). Cardiac-specific overexpression of caveolin-3 preserves t-tubular ICa during heart failure in mice. Exp Physiol.

[25] Körner A, Mosqueira M, Hecker M, Ullrich ND (2021). Substrate stiffness influences structural and functional remodeling in induced pluripotent stem cell-derived cardiomyocytes. Front Physiol.

[26] Lakatta EG, DiFrancesco D (2009). What keeps us ticking: a funny current, a calcium clock, or both?. J Mol Cell Cardiol.

[27] Lemoine MD, Mannhardt I, Breckwoldt K, Prondzynski M, Flenner F, Ulmer B, Hirt MN, Neuber C, Horváth A, Kloth B, Reichenspurner H, Willems S, Hansen A, Eschenhagen T (2017). Christ T (2017) Human iPSC-derived cardiomyocytes cultured in 3D engineered heart tissue show physiological upstroke velocity and sodium current density. Sci Reports.

[28] Lian X, Zhang J, Azarin SM, Zhu K, Hazeltine LB, Bao X, Hsiao C, Kamp TJ, Palecek SP (2013). Directed cardiomyocyte differentiation from human pluripotent stem cells by modulating Wnt/β-catenin signaling under fully defined conditions. Nat Protoc.

[29] Lieu DK, Liu J, Siu CW, McNerney GP, Tse HF, Abu-Khalil A, Huser T, Li RA (2009). Absence of transverse tubules contributes to non-uniform Ca2+ wavefronts in mouse and human embryonic stem cell-derived cardiomyocytes. Stem Cells Dev.

[30] Liu C, Spinozzi S, Chen JY, Fang X, Feng W, Perkins G, Cattaneo P, Guimarães-Camboa N, Dalton ND, Peterson KL, Wu T, Ouyang K, Fu XD, Evans SM, Chen J (2019). Nexilin is a new component of junctional membrane complexes required for cardiac T-tubule formation. Circulation.

[31] Liu Y, Zhou K, Li J, Agvanian S, Caldaruse AM, Shaw S, Hitzeman TC, Shaw RM, Hong TT (2020). In mice subjected to chronic stress, exogenous cBIN1 preserves calcium-handling machinery and cardiac function. JACC Basic to Transl Sci.

[32] Louch WE, Mørk HK, Sexton J, Strømme TA, Laake P, Sjaastad I, Sejersted OM (2006). T-tubule disorganization and reduced synchrony of Ca2+ release in murine cardiomyocytes following myocardial infarction. J Physiol.

[33] Lundy SD, Zhu WZ, Regnier M, Laflamme MA (2013). Structural and functional maturation of cardiomyocytes derived from human pluripotent stem cells. Stem Cells Dev.

[34] Maltsev VA, Lakatta EG (2008). Dynamic interactions of an intracellular Ca2+ clock and membrane ion channel clock underlie robust initiation and regulation of cardiac pacemaker function. Cardiovasc Res.

[35] McDevitt TC, Angello JC, Whitney ML, Reinecke H, Hauschka SD, Murry CE, Stayton PS (2002). In vitro generation of differentiated cardiac myofibers on micropatterned laminin surfaces. J Biomed Mater Res.

[36] Parikh SS, Blackwell DJ, Gomez-Hurtado N, Frisk M, Wang L, Kim K, Dahl CP, Fiane A, Tønnessen T, Kryshtal DO, Louch WE, Knollmann BC (2017). Thyroid and glucocorticoid hormones promote functional T-tubule development in human-induced pluripotent stem cell-derived cardiomyocytes. Circ Res.

[37] Poulet C, Sanchez-Alonso J, Swiatlowska P, Mouy F, Lucarelli C, Alvarez-Laviada A, Gross P, Terracciano C, Houser S, Gorelik J (2021). Junctophilin-2 tethers T-tubules and recruits functional L-type calcium channels to lipid rafts in adult cardiomyocytes. Cardiovasc Res.

[38] Prokic I, Cowling BS, Kutchukian C, Kretz C, Tasfaout H, Gache V, Hergueux J, Wendling O, Ferry A, Toussaint A, Gavriilidis C, Nattarayan V, Koch C, Lainé J, Combe R, Tiret L, Jacquemond V, Pilot-Storck F, Laporte J (2020). Differential physiological roles for BIN1 isoforms in skeletal muscle development, function and regeneration. DMM Dis Model Mech.

[39] Prokic I, Cowling BS, Laporte J (2014). Amphiphysin 2 (BIN1) in physiology and diseases. J Mol Med.

[40] Rao C, Prodromakis T, Kolker L, Chaudhry UAR, Trantidou T, Sridhar A, Weekes C, Camelliti P, Harding SE, Darzi A, Yacoub MH, Athanasiou T, Terracciano CM (2013). The effect of microgrooved culture substrates on calcium cycling of cardiac myocytes derived from human induced pluripotent stem cells. Biomaterials.

[41] Razzaq A, Robinson IM, McMahon HT, Skepper JN, Su Y, Zelhof AC, Jackson AP, Gay NJ, O’Kane CJ (2001). Amphiphysin is necessary for organization of the excitation-contraction coupling machinery of muscles, but not for synaptic vesicle endocytosis in Drosophila. Genes Dev.

[42] Regev D, Baskin P, Dolgopyat I, Davidor M, Kermani F, Ullrich ND, Binah O (2021). Induced pluripotent stem cell-derived cardiomyocytes: generation and enrichment protocols, immature and mature structure and function. Recent advances in IPSC-derived cell types: volume 4 in advances in stem cell biology.

[43] Ribeiro AJS, Ang YS, Fu JD, Rivas RN, Mohamed TMA, Higgs GC, Srivastava D, Pruitt BL (2015). Contractility of Single cardiomyocytes differentiated from pluripotent stem cells depends on physiological shape and substrate stiffness. Proc Natl Acad Sci U S A.

[44] Rössler U, Hennig AF, Stelzer N, Bose S, Kopp J, Søe K, Cyganek L, Zifarelli G, Ali S, von der Hagen M, Strässler ET, Hahn G, Pusch M, Stauber T, Izsvák Z, Gossen M, Stachelscheid H, Kornak U (2021). Efficient generation of osteoclasts from human induced pluripotent stem cells and functional investigations of lethal CLCN7-related osteopetrosis. J Bone Miner Res.

[45] Ruan JL, Tulloch NL, Razumova MV, Saiget M, Muskheli V, Pabon L, Reinecke H, Regnier M, Murry CE (2016). Mechanical stress conditioning and electrical stimulation promote contractility and force maturation of induced pluripotent stem cell-derived human cardiac tissue. Circulation.

[46] Silbernagel N, Körner A, Balitzki J, Jaggy M, Bertels S, Richter B, Hippler M, Hellwig A, Hecker M, Bastmeyer M, Ullrich ND (2020). Shaping the heart: Structural and functional maturation of iPSC-cardiomyocytes in 3D-micro-scaffolds. Biomaterials.

[47] Singh JK, Barsegyan V, Bassi N, Marszalec W, Tai S, Mothkur S, Mulla M, Nico E, Shiferaw Y, Aistrup GL, Wasserstrom JA (2017). T-tubule remodeling and increased heterogeneity of calcium release during the progression to heart failure in intact rat ventricle. Physiol Rep.

[48] Snir M, Kehat I, Gepstein A, Coleman R, Itskovitz-Eldor J, Livne E, Gepstein L (2003). Assessment of the ultrastructural and proliferative properties of human embryonic stem cell-derived cardiomyocytes. Am J Physiol Heart Circ Physiol.

[49] Sun XH, Protasi F, Takahashi M, Takeshima H, Ferguson DG, Franzini- Armstrong C (1995). Molecular architecture of membranes involved in excitation-contraction coupling of cardiac muscle. J Cell Biol.

[50] Takeshima H, Komazaki S, Nishi M, Iino M, Kangawa K (2000). Junctophilins: A novel family of junctional membrane complex proteins. Mol Cell.

[51] Tiburcy M, Hudson JE, Balfanz P, Schlick S, Meyer T, Liao MLC, Levent E, Raad F, Zeidler S, Wingender E, Riegler J, Wang M, Gold JD, Kehat I, Wettwer E, Ravens U, Dierickx P, Van Laake LW, Goumans MJ, Khadjeh S, Toischer K, Hasenfuss G, Couture LA, Unger A, Linke WA, Araki T, Neel B, Keller G, Gepstein L, Wu JC, Zimmermann WH (2017). Defined engineered human myocardium with advanced maturation for applications in heart failure modeling and repair. Circulation.

[52] Tjondrokoesoemo A, Park KH, Ferrante C, Komazaki S, Lesniak S, Brotto M, Ko JK, Zhou J, Weisleder N, Ma J (2011). Disrupted membrane structure and intracellular Ca2+ signaling in adult skeletal muscle with acute knockdown of bin1. PLoS ONE.

[53] Tsien RW, Kass RS, Weingart R (1979). Cellular and subcellular mechanisms of cardiac pacemaker oscillations. J Exp Biol.

[54] Wang PY, Yu J, Lin JH, Tsai WB (2011). Modulation of alignment, elongation and contraction of cardiomyocytes through a combination of nanotopography and rigidity of substrates. Acta Biomater.

[55] Wei S, Guo A, Chen B, Kutschke W, Xie YP, Zimmerman K, Weiss RM, Anderson ME, Cheng H, Song LS (2010). T-tubule remodeling during transition from hypertrophy to heart failure. Circ Res.

[56] Yamaguchi Y, Iribe G, Nishida M, Naruse K (2017). Role of TRPC3 and TRPC6 channels in the myocardial response to stretch: linking physiology and pathophysiology. Prog Biophys Mol Biol.

[57] Yamakawa S, Wu D, Dasgupta M, Pedamallu H, Gupta B, Modi R, Mufti M, OCallaghan C, Frisk M, Louch WE, Arora R, Shiferaw Y, Burrell A, Ryan J, Nelson L, Chow M, Shah SJ, Aistrup G, Zhou J, Marszalec W, Andrew Wasserstrom J (2021). Role of t-tubule remodeling on mechanisms of abnormal calcium release during heart failure development in canine ventricle. Am J Physiol Heart Circ Physiol.

[58] Yang X, Pabon L, Murry CE, Wang G, Jacquet L, Karamariti E, Xu Q, Santillo M, Colantuoni A, Mondola P, Guida B, Damiano S, Montezano AC, Touyz RM, Matsa E, Burridge PW, Yu KH, Ahrens JH, Termglinchan V, Wu H, Liu C, Shukla P, Sayed N, Churko JM, Shao N, Woo NA, Chao AS, Gold JD, Karakikes I, Snyder MP, Wu JC, Xu C, Wang L, Yu Y, Yin F, Zhang X, Jiang L, Qin J, Paravicini TM, Touyz RM, Murray TVA, Smyrnias I, Shah AM, Brewer AC, Montezano AC, Burger D, Paravicini TM, Chignalia AZ, Yusuf H, Almasri M, He Y, Callera GE, He G, Krause KH, Lambeth D, Quinn MT, Touyz RM, Mancini SJ, White AD, Bijland S, Rutherford C, Graham D, Richter EA, Viollet B, Touyz RM, Palmer TM, Salt IP, Magder S, Lotufo PA, Pereira AC, Vasconcellos PS, Santos IS, Mill JG, Bensenor IM, Lambeth JD, Sedeek M, Hébert RL, Kennedy CR, Burns KD, Touyz RM, Ji H, Kim HSHS, Kim HSHS, Leong KW, Kim HSHS, Leong KW, Cheung C, Bernardo AS, Pedersen RA, Sinha S, Cheung BMY, Li C, Biel NM, Santostefano KE, DiVita BB, El RN, Carrasquilla SD, Simmons C, Nakanishi M, Cooper-DeHoff RM, Johnson JA, Terada N, Amar L, Sharabi Y, Rossi GP, Vidal-Petiot E, Dominiczak AF, Mulatero P, Faucon AL, Dhaun N, Touyz RM, Barigou M, Lorthioir A (2014). Engineering adolescence: maturation of human pluripotent stem cell-derived cardiomyocytes Xiulan. Circ Res.

[59] Yang X, Rodriguez ML, Leonard A, Sun L, Fischer KA, Wang Y, Ritterhoff J, Zhao L, Kolwicz SC, Pabon L, Reinecke H, Sniadecki NJ, Tian R, Ruohola-Baker H, Xu H, Murry CE (2019). Fatty acids enhance the maturation of cardiomyocytes derived from human pluripotent stem cells. Stem Cell Reports.

[60] Yu Z-Y, Gong H, Kesteven S, Guo Y, Wu J, Li JV, Cheng D, Zhou Z, Iismaa SE, Kaidonis X, Graham RM, Cox CD, Feneley MP, Martinac B (2022). Piezo1 is the cardiac mechanosensor that initiates the cardiomyocyte hypertrophic response to pressure overload in adult mice. Nat Cardiovasc Res.

[61] Zhang X, hua, Morad M,  (2020). Ca2+ signaling of human pluripotent stemcells-derived cardiomyocytes as compared to adult mammaliancardiomyocytes. Cell Calcium.

[62] Zhao M, Tang Y, Zhou Y, Zhang J (2019). Deciphering role of wnt signalling in cardiac mesoderm and cardiomyocyte differentiation from human iPSCs: four-dimensional control of wnt pathway for hiPSC-CMs differentiation. Sci Rep.

[63] Zhou K, Agvanian S, Liu Y, Hitzeman T, Shaw RM, Hong T (2018). Abstract 305: AAV9 mediated cardiac bin1 gene therapy attenuates pressure overload-induced heart failure in mice. Circ Res.

