# Supplementary material for: Membrane remodelling triggers maturation of excitation–contraction coupling in 3D-shaped human-induced pluripotent stem cell-derived cardiomyocytes
Source: Basic Res Cardiol. 2023 Mar 29;118(1):13. doi: 10.1007/s00395-023-00984-5 (PMC10060306; doi:10.1007/s00395-023-00984-5)
Supplement: Supplementary file 1 — Supplementary file1 (DOCX 765 KB) [file 395_2023_984_MOESM1_ESM.docx]

**Supplemental Information**

**Title:**

Membrane remodelling triggers maturation of excitation-contraction coupling in 3D-shaped human induced pluripotent stem cell-derived cardiomyocytes

**Authors:**

Fatemeh Kermani^1^, Matias Mosqueira^1^, Kyra Peters^1^, Enrico D. Lemma^2,§^, Kleopatra Rapti^3^, Dirk Grimm^3,4,5^, Martin Bastmeyer^2,6,7^, Magdalena Laugsch^8^, Markus Hecker^1,4^, Nina D. Ullrich^1,4,7,9*^

**Affiliations:**

^1^Department of Cardiovascular Physiology, Heidelberg University, Heidelberg, Germany;

^2^Zoological Institute, Cell and Neurobiology, Karlsruhe Institute of Technology (KIT), Karlsruhe, Germany;

^§^Current address: Department of Engineering, Università Campus Bio-Medico di Roma, Rome, Italy;

^3^Department of Infectious Diseases/Virology, Section Viral Vector Technologies, Heidelberg University, Heidelberg, Germany;

^4^German Center for Cardiovascular Research (DZHK), Partner Site Heidelberg/Mannheim, Heidelberg, Germany;

^5^German Center for Infection Research (DZIF), Partner Site Heidelberg, Germany;

^6^Institute of Biological and Chemical Systems – Biological information processing (IBCS-BIP), Karlsruhe Institute of Technology (KIT), Eggenstein-Leopoldshafen, Germany;

^7^Heidelberg-Karlsruhe Research Partnership (HEiKA), Research Bridge (Synthetic Biology), Heidelberg University and Karlsruhe Institute of Technology, Germany;

^8^Institute of Human Genetics, Heidelberg University, Heidelberg, Germany;

^9^Lead contact.

**Expanded Methods**

1. **Culture and maintenance of hiPSC**

All experiments were conducted using hiPSC, kindly provided by Dr. Lukas Cyganek, Stem Cell Unit Göttingen, University Medical Center Göttingen (UMG). Wild type iPSC line UMGi014-C clone 14 (isWT1.14) was generated from dermal fibroblasts using the integration-free Sendai virus and described previously (Rössler *et al.*, 2021). Two other cell lines were used to replicate the representative experiments (ethical approval number for all hiPSC lines used in this study: S-455/2018). Standard cell culture plates (6- and 12-well-plates, SARSTEDT, Nümbrecht, Germany), were coated with Matrigel (Corning, Berlin, Germany) (1:300 in DMEM/F-12, Gibco, Darmstadt, Germany) and incubated for at least 30 minutes at 37°C with 5% CO_2_. hiPSC with 70% confluency were sub-cultured at a ratio of 1:7 (12-well-plate) and 1:14 (6-well-plate) for expansion and differentiation purposes, respectively. Briefly, cells were rapidly rinsed twice with PBS (Sigma-Aldrich, Darmstadt, Germany) and incubated in 0.5 M ethylenediamine tetraacetic acid (EDTA, Thermo Fisher scientific, Darmstadt, Germany) for 7 minutes. Cells were detached using StemFit Basic04 Complete Medium (Nippon Genetics, Düren, Germany) containing the ROCK-inhibitor thiazovivin (1:1000, Stemcell Technologies, Cologne, Germany). The cell suspension was centrifuged at 400 rpm for 5 mins and the pellet was resuspended and replated in freshly coated wells. After 24 h, the medium was changed to StemFit medium without thiazovivin. The medium was refreshed every 2 days until the desired confluency was reached. Cells were either passaged or cardiogenic differentiation was initiated.

1. **Differentiation of hiPSC into cardiomyocytes**

hiPSC were differentiated into cardiomyocytes by modulation of the Wnt/β-catenin signalling pathway at 95-100% confluency (Zhao *et al.*, 2019). To initiate differentiation, cells were treated with GSK3β inhibitor CHIR99021 (5 µM, Stemcell Technologies) in RPMI 1640 containing B-27 supplements without insulin (Gibco) to activate the Wnt signalling pathway (day 0). After 24 h, cells were maintained in 2.5 µM CHIR99021 to improve differentiation efficiency (Zhao *et al.*, 2019). On day 3, cells were incubated with the Wnt-inhibitor IWP-4 (5 µM Stemcell Technology, in RPMI plus B-27 without insulin). On day 5, medium was changed to RPMI 1640 plus B27 without insulin, and on day 7 cells were cultivated in RPMI plus B-27 with insulin. Differentiated cardiomyocytes started to contract on day 9. Thereafter, cells were purified by metabolic selection (Gibco), where glucose was replaced by sodium–lactate (Sigma-Aldrich) for 4-5 days to prevent growth of non-cardiomyocytes. Finally, cells were maintained in RPMI plus B27 with insulin until further experiments.

1. **hiPSC-CM dissociation and culture**

Between days 15-20 post differentiation, hiPSC-CM were dissociated for experimental purposes. Briefly, cells were rinsed twice with PBS and incubated with TrypLE Express (1-5x, Gibco) at 37°C and 5% CO_2_ for 10 minutes. Detached cells were transferred to splitting medium (RPMI plus B27 with insulin, 10% FBS, Gibco, 0.1% thiazovivin and 1% penicillin/streptomycin, Sigma-Aldrich) to quench the enzyme activity. The cell suspension was centrifuged at 200 rpm for 10 minutes and the pellet was resuspended in 2-4 ml of splitting medium. Cells were counted using a hemacytometer and seeded at a density of 50−70 and 20−30×10^3^ cells on 3D-micro-scaffolds and conventional glass bottom dishes (MatTek Europe, Bratislava, Slovakia), respectively. After 24 h medium was changed to RPMI plus B-27 with insulin.

1. **3D-micro-scaffold fabrication**

3D-micro-scaffolds were constructed via two-photon lithography at the Karlsruhe Institute of Technology (KIT) using commercially available equipment (Photonic Professional GT2 from Nanoscribe GmbH). Briefly, two-photon lithography is a direct laser writing technique (DLW) to realize 3D micro-scaffolds at a maximum resolution below 100 nm: it exploits the polymerization of a photosensitive material as a consequence of the exposure to a focused, femtosecond-pulsed laser (Hippler *et al.*, 2019). In fact, a liquid droplet of photoresist (pentaerythritol triacrylate (PETA, Sigma-Aldrich) with 2% w/w of the photoinitiator Irgacure 819 (BASF, Ludwigshafen, Germany)) was firstly placed on a 25x25 mm silanized glass coverslip. Upon exposure, the material started to polymerize, while the deflection of the laser beam via galvanometric mirrors and the relative movement of the substrate allowed to fabricate solid 3D structures in the shapes of cuboids (22 µm × 55 µm × 20 µm length x width x height) and hexagons (side 22 µm, height 20 µm) with a wall thickness of ≈2 µm. Geometries and polymerization parameters (e.g., scanning speed and laser power) were optimized starting from a previous publication (Silbernagel *et al.*, 2020), to obtain stable and stiff scaffolds. After polymerization via two-photon lithography, the unexposed material was developed in isopropanol for 30 min and dried with a stream of nitrogen.

**5. SEM** **imaging**

Cuboid and hexagonal micro-structures were visualized via scanning electron microscopy (SEM). After fabrication via two-photon lithography, development and drying, the structures were sputtered with 7-10 nm of gold. Immediately afterwards, micrographs were taken with a Zeiss Supra SEM, at a voltage of 1-3 kV using a secondary electrons detector.

1. **Production of recombinant AAV6 vectors and cells transduction**

AAV6 vectors containing either the gene for the fluorescent reporter ds-Red alone or a bicistronic construct with BIN1 coupled to dsRed via an IRES were cloned and produced. Briefly, 293T cells were transfected using polyethylenimine (PEI, Polysciences) and the 3-plasmid system (adenoviral helper plasmid, a plasmid containing Rep2 and Cap6 and either of the transgene plasmids). Cells were collected 3 days after transfection, thrice freeze-thawed, and centrifuged (4000 g, 15 minutes). The supernatant was loaded onto iodixanol gradients and centrifuged for 2 h in an OptimaTM L-90K Ultracentrifuge (Beckman Coulter). The fraction containing the AAVs was collected as previously described (Jungmann, Müller and Rapti, 2017). The viral productions were quantified using qPCR in a Step One Plus Real-Time PCR System (Applied Biosystems).

Settled cells were transduced with 1.0×10^5^ genome copies per cell of either AAV6-CMV-ds-Red as a negative control or AAV6-CMV-cBIN1-ds-Red for 24 h.

1. **Immunocytochemistry**

Cultured hiPSC-CM grown in scaffolds were used for immunocytochemistry experiments. Cells were incubated with prewarmed Tyrode solution containing 10 µM blebbistatin (Tocris, Wiesbaden-Nordenstadt, Germany) to inhibit contraction for 10 minutes. Subsequently, cells were fixed with 4% paraformaldehyde (PFA, Thermo Fisher scientific) for 10 minutes at room temperature and washed three times for 5 minutes with PBS. The following samples were permeabilized with 0.1% Triton X (Merck, Darmstadt, Germany) and the unspecific binding sites were blocked with 0.1% Triton X and 1% bovine serum albumin (BSA, Sigma-Aldrich) in PBS at room temperature for 15 and 30 minutes, respectively. Cells were then incubated for 90 minutes with following primary antibodies in blocking buffer: ɑ-actinin (1:400, A7811, Sigma-Aldrich), BIN1 (1:100, PA5-82120, Invitrogen), LTCC (1:50, ACC-003, Alomone Labs), RyR2 (1:100, ab2827, Abcam), NCX (1:400, ANX-011, Alomone Labs), SERCA2 (1:100, AB2861, Abcam). After three times washing with PBS for 5 minutes, samples were exposed to the corresponding secondary antibodies conjugated to Alexa Fluor dyes with different excitation-emission spectra for 1 h: Alexa Fluor 488 goat anti-mouse (1:400, A11001, Life Technologies), Alexa Fluor 488 goat anti-rabbit (1:400, A11008, Life Technologies) and Alexa Fluor 568 donkey anti-rabbit (1:400, A10042, Life Technologies). For actin staining, samples were incubated with Phalloidin-TRITC (1:100, P1951-1MG, Sigma-Aldrich) for 1 h. After threefold washing, cells were mounted with fluoroshield containing DAPI (Sigma-Aldrich) for nuclei staining. Samples were imaged using a laser scanning confocal microscope (Leica TCS SP8 LSCM, Leica Microsystems CMS GmbH, Mannheim, Germany) with a 63x oil immersion objective (NA 1.4) and the acquisition software LAS-X (Vers. 3.5.0.18371, Leica Microsystems).

1. **Live imaging**

The standard bath solution for live imaging and electrophysiological experiments was based on a modified Tyrode’s solution comprising (in mM): NaCl 140, KCl 5.4, CaCl_2_ 1.8, MgCl_2_ 1.1, HEPES 5 and glucose 10 with pH 7.4.

For t-tubule staining, live cells were incubated with 3-4 µM di-8-ANEPPS (Invitrogen) in prewarmed Tyrode for 10 minutes at room temperature in dark. Cells were washed twice with Tyrode to remove unbound dye. 10 µM blebbistatin was added to the Tyrode solution during imaging. Images were taken using a laser-scanning confocal microscope (Olympus FLUOVIEW FV1000) with a 60x water-immersion objective (NA 1.2). 2D images were acquired at a frequency of 40 µs/pixel with 1024 pixels x 1024 lines. Quantitative analysis of t-tubule density was performed using ImageJ/Fiji. First, images were filtered by applying the bandpass filter command and then default threshold was set. Images were binarized and consecutively skeletonized. A 40 µm × 10 µm region of interest (ROIs) excluding surface membrane and nuclei was selected. Finally, the percentage of black pixels per area was calculated to determine t-tubule density.

1. **Proximity ligation assay (PLA)**

To quantify dyadic units formed by Cav1.2 and RyR2 in hiPSC-CM, PLA was performed using Duolink® Proximity Ligation Assay kit (Sigma-Aldrich) by following the manufacturer’s protocol. Briefly, cells were rinsed twice with PBS, fixed with 4% PFA for 10 minutes at room temperature, washed three times for 5 minutes. Thereafter, cells were permeabilized with 0.3% Triton X for 15 minutes and blocked with blocking solution for 1 h in 37°C. Samples were incubated with primary antibodies for the target proteins (mouse-anti-RyR2 and rabbit-anti- Cav1.2) for 24 h at 4°C. After twice washing, cells were incubated with the PLA probes anti-mouse minus and anti-rabbit plus for 1 h at 37°C. After washing, ligation and amplification steps were carried out at at 37°C for 30 and 110 minutes, respectively. Finally, cells were washed and mounted with Duolink In Situ Mounting Medium with DAPI, dried overnight and imaged using the Leica SP8 confocal microscope. To quantify fluorescent signals, images were filtered by applying the bandpass filter command, binarized and the percentage of black pixels per area was calculated to determine dyadic unit density.

1. **Ca^2+^ imaging**

To record spontaneous Ca^2+^ release activity and stimulated Ca^2+^ transients, cells were loaded with 3 µM fluo-4-AM (Life Technology) diluted in Tyrode’s solution for 20−25 minutes at room temperature in dark. Afterwards, cells were maintained in pre-warmed Tyrode for 10 minutes to de-esterify fluo-4. Ca^2+^ transients and Ca^2+^ sparks were recorded along the longitudinal or transversal axis of each cell using the line-scan mode. Further experiments were performed to measure triggered Ca^2+^ transients. Cells were paced with a field stimulator (Myopacer, IonOptix, Dublin, Ireland) at 20 V and 1 Hz and line-scan images were recorded during pacing. The recordings were collected using the LSCM (Olympus Fluoview FV1000, Hamburg, Germany) with a 60x water immersion objective lens. Line-scans were obtained at 4 µs/pixel, 2 ms/line and 5000 lines/images. Fluo-4 was excited at 473 nm and fluorescence emission was collected at 490-545 nm. For line-scan image analysis, a line profile was generated. After background subtraction, the fluorescent signal was normalised to baseline intensity (F/F_0_). The Ca^2+^ transients and Ca^2+^ sparks kinetics were analysed. Ca^2+^ transients measurements comprised time-to-peak (TTP), decay time and full duration at half maximum (FDHM). The decay of Ca^2+^ transients was fitted and calculated with an exponential decay function. Ca^2+^ sparks evaluation included frequency and spatio-temporal properties.

1. **Cellular electrophysiology**

L-type calcium currents (I_CaL_) of hiPSC-CM were measured at physiological temperature (35-37°C) using the whole-cell configuration of the patch-clamp technique (HEKA EPC-10 patch-clamp amplifier, HEKA Elektronik GmbH, Reutlingen, Germany). Global superfusion with fresh Tyrode’s solution was applied to avoid nutrients depletion and temperature was controlled with a heater controller (NPI Electronic GmbH, Tamm, Germany). Borosilicate glass pipettes were pulled and heat polished to obtain patch pipettes with a resistance between 2−6 MΩ using a DMZ Zeitz-puller (Zeitz-Instruments Vertriebs GmbH, Martinsried, Germany). The internal solution contained (in mM) 8 NaCl, 120 CsAsp, 20 TEA-Cl, 5.9 MgCl_2_, 20 HEPES, 5 K_2_-ATP (Sigma-Aldrich), and 0.05 of the Ca^2+^-sensitive fluorescent indicator K_5_-fluo-3 (Life Technology). The external Tyrode’s solution contained 5 mM CsCl to eliminate I_K_.

The current-voltage (I-V) relationship was evaluated by a voltage clamp protocol starting from a holding potential of −80 mV followed by 400 s depolarizing pulses from −40 mV to +50 mV in 10 mV increments (Figure 4A). Ca^2+^ current densities were calculated by dividing the peak current amplitudes by cell membrane capacitance. The steady-state inactivation and activation of I_CaL_ was assessed using a two-step protocol. To measure the voltage-dependent inactivation of I_CaL_, the membrane potential was held at −80 mV followed by a prepulse from −60 to +20 mV with an increment of 10 mV for 700 ms, returned to −40 for 10 ms and then a test pulse of 0 mV for 400 ms (Figure 4D). To determine the voltage-dependence of activation of I_CaL_, voltage was clamped to +70 mV for 700 ms, returned to −40 for 10 ms and followed by a step to −50 mV and looping at an increasing interval of 10 mV up until +10 mV for 400 ms (Figure 4F). To obtain V_1/2_ of inactivation and activation, both curves were fitted with the Boltzmann equation in OriginPro.

To specifically study EC coupling, the EC coupling gain was evaluated by evoking CICR using the whole-cell patch-clamp technique and confocal imaging in the line-scan mode simultaneously. Ca^2+^ currents (I_CaL_) were elicited with a two-step voltage protocol. Starting from a membrane potential of ̶ 80 mV, a 500 ms voltage-ramp to ̶ 40 mV was applied to inactivate the fast voltage-dependent Na^+^ current (I_Na_). After 2 s, the first test step was set to −25 mV for 400 ms to activate I_CaL_ and trigger CICR at low amplitude. The second test step was set to +10 mV to maximally activate I_CaL_; this test step served as a control measurement for CICR. Simultaneously, Ca^2+^ transients elicited by I_CaL_ were recorded and changes in fluorescent intensity were plotted over time. To evaluate the EC coupling gain, the ratio of maximal Ca^2+^ transient and I_CaL_ amplitudes at −25 mV was calculated. For examination of Ca^2+^-dependent inactivation, I_CaL_ inactivation at +10 mV was fitted with a biexponential function in OriginPro.

To investigate fractional release and NCX activity, voltage-clamped cells were paced at 1 Hz for several seconds, then succeeded by local caffeine application (10 mM, RyR2 agonist, Sigma-Aldrich) to deplete the SR Ca^2+^ content. Caffeine-elicited NCX currents were measured at the same time. Fractional release was defined as the ratio of Ca^2+^ transient amplitude during stimulation and the maximal caffeine-induced Ca^2+^ transient amplitude. NCX activity was measured by integration of inward current during caffeine application.

1. **Quantitative RT-PCR**

Total RNA from approximately 1×10^6^ cells was extracted and isolated using RNeasy Mini kit (QIAGEN, Hilden, Germany) and reverse-transcribed into cDNA using the QuantiTect® Reverse Transcription kit (QIAGEN). Gene expression was quantified by the Maxima SYBR green/ROX qPCR master mix (Thermo Fisher Scientific) and quantitative PCR was performed using the 7900HT Fast Real-Time PCR System for 45 cycles. All primer sequences (Sigma-Aldrich) were taken from previously published primer pairs listed in Table 1. Expression levels were normalized to the housekeeping gene PGC-1α. Data were analysed based on fold change in expression (∆∆Ct).

**Table S1:** List of primer sequences used for qRT-PCR.

| **Gene** | **Forward** | **Reverse** |
| --- | --- | --- |
| **PGC1α^1^** | AACACTTACAAGCCAAACCA | GGGTTCAATAGTCTTGTTCTC |
| **BIN1^2^** | ATGAGGCAAACAAGATCGCAG | CGTGACTTGATGTCGGGGAA |
| **cTNT^2^** | ACAGAGCGGAAAAGTGGGAAG | TCGTTGATCCTGTTTCGGAGA |
| **MYH6^1^** | CTTCTCCACCTTAGCCCTGG | GCTGCCCCTTCAACTACAGA |
| **MYH7^1^** | CGCACCTTCTTCTCTTGCTC | GAGGACAAGGTCAACACCCT |
| **ACTN2^1^** | CTGCTGCTTTGGTGTCAGAG | TTCCTATGGGGTCATCCTTG |
| **CACNA1C^1^** | CAATCTCCGAAGAGGGGTTT | TCGCTTCAGACATTCCAGGT |
| **RYR2^1^** | AAGCCCTCTCGTCTGAAACA | CCACCCAGACATTAGCAGGT |
| **ATP2A2^2^** | CATCAAGCACACTGATCCCGT | CCACTCCCATAGCTTTCCCAG |
| **NCX** | GGGACTAACAGCTGGAGAGAA | CCACATTCATCGTCGTCATCA |

^1^ Ribeiro *et al.*, 2015); ^2^ Ronaldson *et al.*, 2018.

1. **Western blot**

Total protein of Ctrl and BIN1-expressing hiPSC-CM was extracted using RIPA buffer containing phosphatase and protease inhibitors (Thermo Fisher Scientific). Lysates were sonicated and centrifuged for 15 minutes at 13000 rpm and 4°C. The supernatant containing whole cell proteins was kept and further processes. Total protein concentration was measured using PierceTM BCA Protein Assay Kit (Thermo Fisher Scientific). Samples containing 20 µg of protein in LDS buffer (lithium dodecyl sulfate, Thermo Fisher Scientific) were denatured for 10 minutes at 70°C. Afterwards, samples were loaded on a 12% polyacrylamide gel (NuPAGETM 12% Bis-Tris Gel, Invitrogen) at 150 V for 2-2.5 h, then transferred to PVDF membrane (invitrogen) at 0.35 A for 45 minutes. The membrane was blocked with TBS-0.1% Tween20 (TBS-T)-5% milk overnight at 4°C. Blots were incubated with the primary antibodies anti-BIN1 (1:500, mouse monoclonal, IgG, Millipore, Darmstadt, Germany) and anti-GAPDH (1:5000, mouse monoclonal, IgG1, Thermo Fisher Scientific) for 2 h at room temperature. The membrane was washed twice with TBS-T and then incubated with HRP-conjugated goat anti-mouse secondary antibodies (1:2000, IgG1, Thermo Fisher Scientific) for 2 h at room temperature. After twice washing with TBS-T, blots were developed with chemiluminescent reagents (PierceTM ECL PlusWestern Blotting Substrate, Thermo Fisher Scientific) and visualized (Intas Science Imaging Instruments GmbH, Germany). Signal intensities were evaluated in ImageJ/Fiji.

1. **Data and statistical analysis**

All images were processed in ImageJ/Fiji. OriginPro® (OriginLab) software was used for all current and Ca^2+^ signal analysis, graphic data design and statistical data analysis. Most of the results were presented as a box plot indicating individual data points, mean (square), median (center horizontal line), interquartile ranges from 25-75% (box edges) and whiskers (standard deviation, SD). qRT-PCR data was presented as a bar graph and whiskers indicated SE. N equals the number of different passage numbers and n shows the number of individually analysed cells. Two-way ANOVA was conducted to evaluate the effect of 3D-reshaping and BIN1 expression on the desired parameters based on our hypothesis followed by Bonferroni post hoc test. Unpaired t-test was used for two groups data comparison. The level of statistical significance is displayed by * and # for p<0.05.

1. **References**

Hippler, M. *et al.* (2019) ‘3D Scaffolds to Study Basic Cell Biology’, *Advanced Materials*, 31(26). doi: 10.1002/adma.201808110.

Jungmann, A., Müller, O. and Rapti, K. (2017) ‘Cell-based measurement of neutralizing antibodies against adeno-associated virus (AAV)’, in *Methods in Molecular Biology*. Methods Mol Biol, pp. 109–126. doi: 10.1007/978-1-4939-6588-5_7.

Ribeiro, A. J. S. *et al.* (2015) ‘Contractility of Single cardiomyocytes differentiated from pluripotent stem cells depends on physiological shape and substrate stiffness’, *Proceedings of the National Academy of Sciences of the United States of America*. National Academy of Sciences, 112(41), pp. 12705–12710. doi: 10.1073/pnas.1508073112.

Ronaldson, K. *et al.* (2018) ‘Advanced maturation of human cardiac tissue grown from pluripotent stem cells’, *Nature*. Springer US. doi: 10.1038/s41586-018-0016-3.

Rössler, U. *et al.* (2021) ‘Efficient generation of osteoclasts from human induced pluripotent stem cells and functional investigations of lethal CLCN7-related osteopetrosis’, *Journal of Bone and Mineral Research*, 36(8), pp. 1621–1635. doi: 10.1002/jbmr.4322.

Silbernagel, N. *et al.* (2020) ‘Shaping the heart: Structural and functional maturation of iPSC-cardiomyocytes in 3D-micro-scaffolds’, *Biomaterials*. Elsevier Ltd, 227, p. 119551. doi: 10.1016/j.biomaterials.2019.119551.

Zhao, M. *et al.* (2019) ‘Deciphering Role of Wnt Signalling in Cardiac Mesoderm and Cardiomyocyte Differentiation from Human iPSCs: Four-dimensional control of Wnt pathway for hiPSC-CMs differentiation’, *Scientific Reports*. Nature Research, 9(1), pp. 1–15. doi: 10.1038/s41598-019-55620-x.

**Supplemental Figures**

**
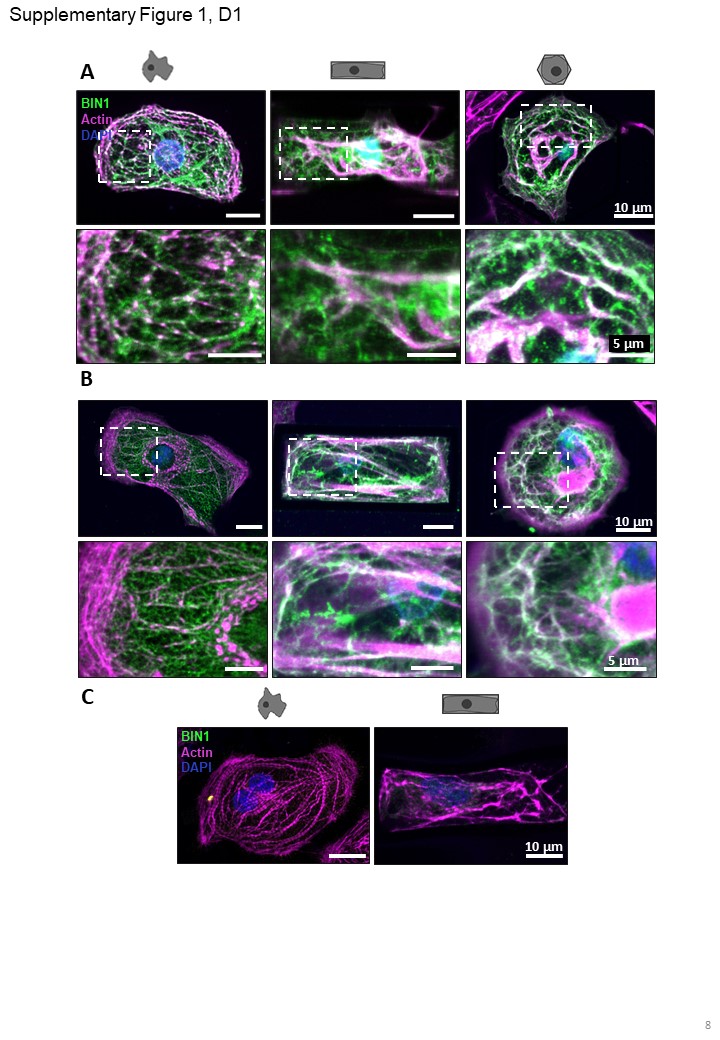
**

**Figure SI. BIN1 expression in two different hiPSC-CM cell lines.** Immunolabelling of BIN1 expression (green), actin (magenta) and DNA (DAPI, blue) **(A, B)** in two different hiPSC-CM lines with different genetic backgrounds transduced with BIN1 in three distinct shapes. **(C)** BIN1-labelling in hiPSC-CM transduced with control AAV6 (without BIN1 expression) in non-patterned and cuboid cells. White boxes indicate the area of magnification.


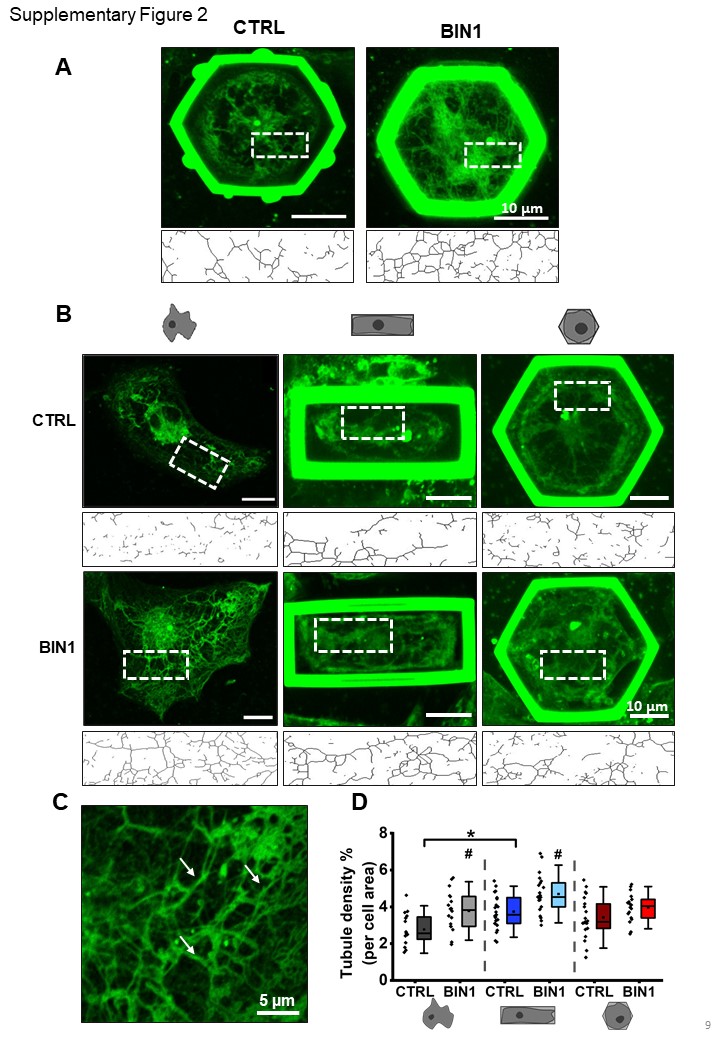


**Figure SII.** **Tubular network biogenesis is induced by BIN1 expression and 3D-reshaping in a different hiPSC-CM cell line.** Representative images of confocal live cell imaging of the plasma membrane using di-8-ANEPPS to depict the tubular network in **(A)** hexagonally shaped hiPSC-CM and **(B)** in second cell line of hiPSC-CM with another genetic background. White boxes show the skeletonized images of the region of interest (ROI). **(C)** Detailed image of the tubular network. **(D)** Statistical analysis of the ROI tubule densities in different experimental groups of hiPSC-CM. Two-way ANOVA was applied; N=3, n=16-23 cells. * indicates comparison between different shapes; # indicates comparison between BIN1-expressing and control cells different shapes; p<0.05. Data are presented as a box plot and whiskers show SD.


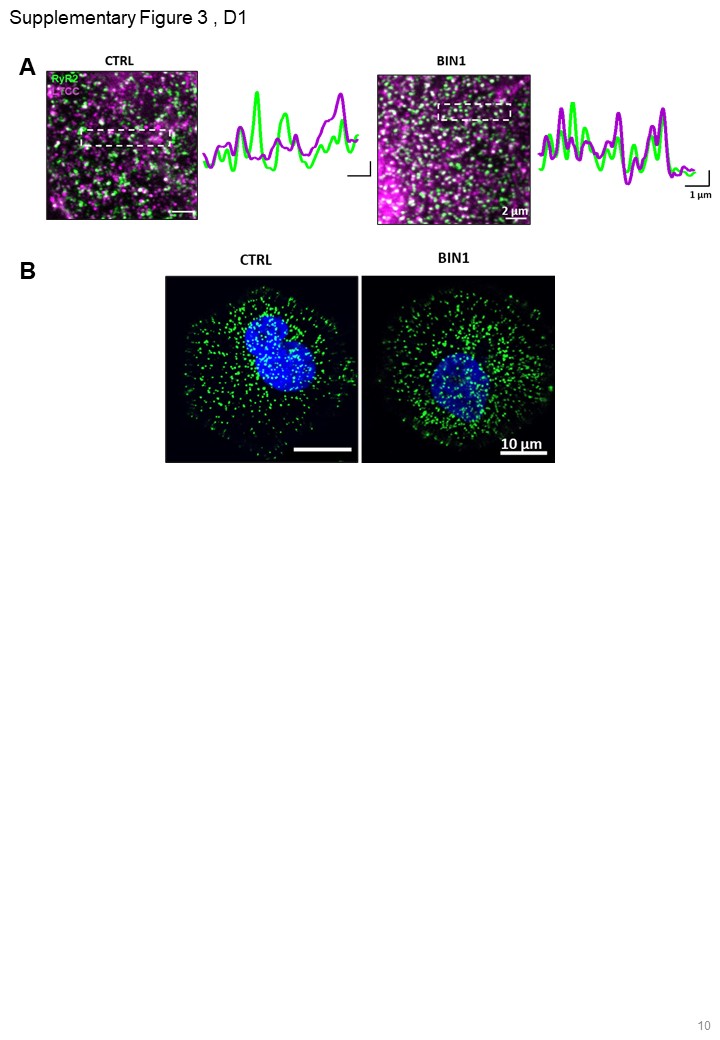


**Figure SIII.** **Co-localization of LTCC and RYR in dyad-like microdomains in hexagonally shaped hiPSC-CM.** **(A)** Immunolabelling of RYR2 (green) and LTCC (magenta) and corresponding intensity profiles from ROIs (white boxes) demonstrating no parallel alignment of both ion channels relative to each other. **(B)** Representative confocal images of the PLA: green signals indicate the sites of interaction between RYR2 and LTCC in dyadic microdomains. Nuclei are stained in blue.


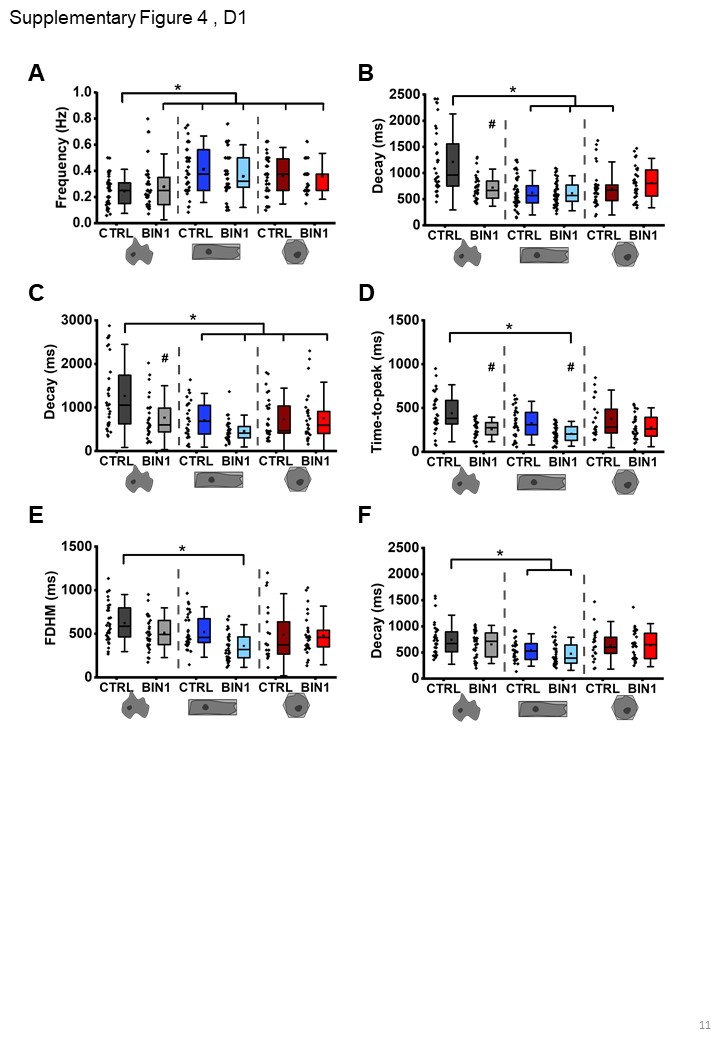


**Figure SIV.** **Spontaneous and triggered Ca^2+^ transients in reshaped and BIN1-expressing hiPSC-CM.** Analysis of spontaneous Ca^2+^ transient frequency **(A)**, decay time of spontaneous **(B)** and stimulated **(C)** Ca^2+^ transients. Analysis of time-to-peak (TTP; **D**), full duration half maximum (FDHM; **E**) and decay time **(F)** of spontaneous Ca^2+^ transients in another cell line of hiPSC-CM with a different genetic background. Statistical differences were tested by two-way ANOVA. N=3, n=21-33 cells. * indicates comparison between different shapes; # indicates comparison between BIN1-overexpressing and shape control cells; p<0.05. Data are presented as a box plot and whiskers show SD.


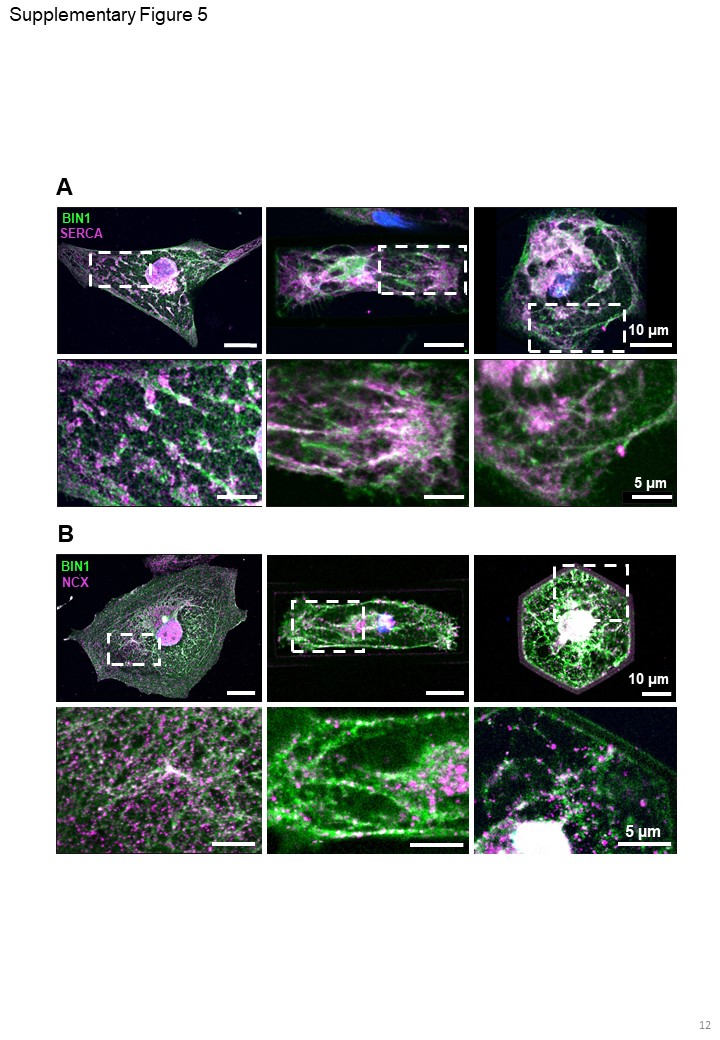


**Figure SV.** **SERCA and NCX expression in reshaped and BIN1-expressing hiPSC-CM.** Immunolabelling of BIN1 (green), SERCA (magenta, A), NCX (magenta, B) and DNA (DAPI, blue) in BIN1-expressing, non-patterned, cuboid and hexagonally-shaped hiPSC-CM. White boxes display the area of magnification.
